# Diversity in genetic risk of recurrent stroke: a genome-wide association study meta-analysis

**DOI:** 10.3389/fstro.2024.1338636

**Published:** 2024-02-21

**Authors:** Chad M. Aldridge, Nicole D. Armstrong, N. Abimbola Sunmonu, Christopher Becker, Deepak Palakshappa, Arne G. Lindgren, Annie Pedersen, Tara M. Stanne, Christina Jern, Jane Maguire, Fang-Chi Hsu, Keith L. Keene, Michele Sale, Marguerite R. Irvin, Bradford B. Worrall

**Affiliations:** ^1^Department of Neurology, University of Virginia, Charlottesville, VA, United States; ^2^Department of Epidemiology, University of Alabama Birmingham, Birmingham, AL, United States; ^3^Department of Neurology, Yale University School of Medicine, Yale University, New Haven, CT, United States; ^4^Department of Neurology, Michigan University, Ann Arbor, MI, United States; ^5^Department of Internal Medicine, Wake Forest University School of Medicine, Winston-Salem, NC, United States; ^6^Department of Clinical Sciences Lund, Neurology, Lund University, Lund, Sweden; ^7^Department of Neurology, Skåne University Hospital Lund, Lund, Sweden; ^8^Department of Laboratory Medicine, Institute of Biomedicine, Sahlgrenska Academy, University of Gothenburg, Gothenburg, Sweden; ^9^Department of Clinical Genetics and Genomics, Region Västra Götaland, Sahlgrenska University Hospital, Gothenburg, Sweden; ^10^Faculty of Health, School of Nursing and Midwifery, University of Technology Sydney, Sydney, NSW, Australia; ^11^Department of Biostatistics and Data Science, Division of Public Health Sciences, Wake Forest University School of Medicine, Winston-Salem, NC, United States; ^12^Department of Genetics, University of Wake Forest, Winston-Salem, NC, United States; ^13^Center for Public Health Genomics, University of Virginia, Charlottesville, VA, United States; ^14^Department of Public Health Sciences, University of Virginia, Charlottesville, VA, United States

**Keywords:** recurrent stroke, genetic risk, diversity, GWAS, African ancestry, meta-analysis, polygenic risk score

## Abstract

**Introduction:**

Stroke is a leading cause of death and disability worldwide. Recurrent strokes are seven times more lethal than initial ones, with 54% leading to long-term disability. Substantial recurrent stroke risk disparities exist among ancestral groups. Notably, Africans face double the risk and higher fatality rates compared to Europeans. Although genetic studies, particularly GWAS, hold promise for uncovering biological insights into recurrent stroke, they remain underexplored. Our study addresses this gap through meta-analyses of recurrent stroke GWAS, considering specific ancestral groups and a combined approach.

**Methods:**

We utilized four independent study cohorts for African, European, and Combined ancestry recurrent stroke GWAS with genotyping, imputation, and strict quality control. We harmonized recurrent stroke phenotype and effect allele estimates across cohorts. The logistic regression GWAS model was adjusted for age, sex, and principal components. We assessed how well genetic risk of stroke informs recurrent stroke risk using Receiver Operating Characteristic (ROC) curve analysis with the GIGASTROKE Consortium's polygenic risk scores (PRS).

**Results:**

Harmonization included 4,420 participants (818 African ancestry and 3,602 European ancestry) with a recurrent stroke rate of 16.8% [median age 66.9 (59.1, 73.6) years; 56.2% male]. We failed to find genome-wide significant variants (*p* < 5e−8). However, we found 18 distinct suggestive (*p* < 5e−6) genetic loci with high biological relevance consistent across African and European ancestries, including PPARGC1B, CCDC3, OPRL1, and MYH11 genes. These genes affect vascular stenosis through constriction and dilation. We also observed an association with SDK1 gene, which has been previous linked with hypertension in Nigerian and Japanese populations). ROC analysis showed poor performance of the ischemic stroke PRS in discriminating recurrent stroke status (area under the curve = 0.48).

**Discussion:**

Our study revealed genetic associations with recurrent stroke not previously associated with incident ischemic stroke. We found suggestive associations in genes previously linked with hypertension. We also determined that knowing the genetic risk of incident stroke does currently not inform recurrent stroke risk. We urgently need more studies to understand better the overlap or lack thereof between incident and recurrent stroke biology.

## 1 Introduction

Stroke is a leading cause of death and disability worldwide, with presentations and mechanisms of action that are quite heterogeneous. In the United States, the majority, nearly 87%, of the ~800,000 strokes that occur each year are ischemic, and a substantial number, roughly 185,000, are recurrent attacks (Benjamin et al., 2019).

Compared to first strokes, recurrent strokes are more deadly and more likely to cause disability. The 30-day case fatality for the first stroke and recurrent stroke is 2 and 14%, respectively (Jong et al., 2004). Likewise, 54% of individuals suffering a recurrent stroke will be disabled (Hankey et al., 2007). In addition to geographical differences in stroke incidence and prevalence, stroke disparities by race-ethnicity are alarming. Stroke has one of the starkest health disparities in the United States. African Americans (AA) endure a nearly 2-fold greater risk of stroke than European Americans (EA; Howard et al., 2016a). AA are also 2–3 times more likely to die from stroke (Howard et al., 2016b). Even among individuals who survive an initial stroke, AA have a much greater risk of stroke recurrence (Sheinart et al., 1998; Park and Ovbiagele, 2016). Considering these facts, it is imperative to investigate the role of genetics in stroke recurrence.

While genome-wide association studies (GWAS) have identified loci and pathways associated with ischemic stroke, such studies on recurrent stroke are limited. It remains unknown what impact genetic ancestry may have on an individual's risk of recurrent stroke. To address these gaps, we conducted a meta-analysis of GWAS data from four independent cohorts to explore general and ancestry-specific genetic contributors to recurrent stroke. Our findings aim to uncover novel genetic variants causally linked to recurrent stroke mechanisms, including indirect mechanisms. Ancestry-specific meta-analyses may provide insights into the genetic factors contributing to recurrent stroke risk disparities between African and European ancestral groups.

## 2 Materials and methods

### 2.1 Study design

This study is a meta-analysis of recurrent stroke genome-wide association study summary statistics from four independent cohorts. Each study included adults aged 18 years or older that had an ischemic stroke that qualified them for study enrollment. The participants were followed until recurrent stroke event or length of the cohort-specific observable period. Each cohort followed participants prospectively. Below, we discuss the cohorts and further detail can be found in Supplementary material 1.

### 2.2 Cohorts

We utilized the following four independent cohorts for this study because of their prospective observation of stroke survivors and their incidence of recurrent stroke events over time: the Australian Stroke Genetics Collaboration (ASGC; Lemmens et al., 2010), the Reasons for Geographic and Racial Differences in Stroke (REGARDS; Howard et al., 2005), the Sahlgrenska Academy Study on Ischemic Stroke (SAHLSIS; Jood et al., 2005), and the Vitamin Intervention for Stroke Intervention clinical trial (VISP; Toole, 2002). Additionally, these cohorts allow the investigation of ancestry-specific genetic associations of recurrent stroke, African and European. The REGARDS and VISP cohorts have notable proportions of African ancestry, while the ASGC and SAHLSIS cohorts are European.

The Australian data comprises 1,230 ischemic stroke cases derived from four acute stroke centers in New South Wales, South Australia and Perth, Western Australia under the banner of the ASGC. These stroke cases comprised European-ancestry stroke patients admitted to clinical centers across Australia. Cases were consecutively recruited from the Neurosciences Department at Gosford Hospital, Gosford, New South Wales (NSW) and the Neurology Department at John Hunter Hospital, Newcastle, NSW between 2003 and 2008. In South Australia, cases were recruited from the Queen Elizabeth Hospital, Adelaide at the same time. For Royal Perth Hospital site, this population-based, longitudinal cohort study sought to determine the age and sex-specific incidence and case fatality of stroke and included residents from the Perth metropolitan area who had a stroke or transient ischemic attack (TIA) between 20 February 1989 and 19 August 1990 (Hankey et al., 2007; Maguire et al., 2011).

The REGARDS study is a population-based, longitudinal cohort comprising 30,239 non-Hispanic Black and White American adults 45 years or older from the 48 contiguous United States and the District of Columbia (Howard et al., 2005). By design, participants were oversampled if they were Black or if they were residents of the stroke belt. The purpose of REGARDS is to investigate the reasons why stroke mortality is higher among Black compared with White adults and residents of the Southeastern US compared with other regions. Enrollment took place in 2003–2007, and participants completed a computer-assisted telephone interview to collect demographic information, as well as an in-home visit for blood pressure measurements and collection of biologic specimens (e.g., blood, urine; Howard et al., 2005).

The SAHLSIS is a hospital-based, longitudinal cohort study, which has been described in detail elsewhere (Jood et al., 2005). In brief, patients with first-ever or recurrent acute ischemic stroke were consecutively recruited at stroke units in Western Sweden. Ischemic stroke cases were aged 18–69 years and were recruited between 1998 and 2011. Ischemic stroke was defined as an episode of focal brain dysfunction with acute onset, lasting >24 h, and of presumed vascular cause with no signs of hemorrhage on neuroimaging. Participants were excluded if further evaluation showed another etiology than stroke.

The VISP trial, a randomized double-blinded trial, has been described previously (Toole, 2002). In summary, the trial investigated the effect of vitamin supplementation dosage on the risk of recurrent stroke. The study enrolled participants with a non-disabling ischemic stroke (mRS ≤ 3) ≥ 72 h before enrollment. Participants (*n* = 3,680) were randomized to a high-dose or low-dose vitamin supplementation arm and reassessed every 3 months until a recurrent stroke event, but not longer than 2 years. Ten sites were not approved for genetic studies, resulting in a subset of 2,100 genotyped participants. There was no intervention effect between treatment groups, thus we considered participants from each treatment group as the same cohort.

### 2.3 Genotyping and quality control

Each cohort underwent genotyping and strict quality control procedures. Supplementary material 1 details the methods by cohort. The genotyping arrays utilized by cohort were the following: ASGC utilized the Illumina HumanHap610-Quad array. REGARDS used the Illumina Infinium Multi-Ethnic AMR/AFR Extended BeadChip arrays. The SAHLSIS performed genotyping on the Illumin Human OmniExpressExome BeadChip version 1.0 or 1.1, while VISP used the Illumina HumanOmni1-Quad-v1 array.

The cohorts had their genotype data referenced to varying genome builds. We utilized the UCSC LiftOver software (Lee et al., 2022) to bring the ASGC, SAHLSIS, and VISP cohorts to the genome assembly GRChr38. We also harmonized the beta coefficients so that the effect allele remained consistent among the cohorts.

### 2.4 Data harmonization

#### 2.4.1 Phenotyping

Recurrent stroke phenotyping definitions by cohort are described here:

ASGC defined stroke by WHO criteria as a sudden focal neurologic deficit of vascular origin, lasting more than 24 h and confirmed by imaging such as computerized tomography (CT) and/or magnetic resonance imaging (MRI) brain scan. Other investigative tests such as electrocardiogram, carotid doppler and trans-esophageal echocardiogram were conducted to define ischemic stroke mechanism as clinically appropriate. Cases were excluded from participation if aged < 18 years, diagnosed with hemorrhagic stroke or transient ischemic attack rather than ischemic stroke, or were unable to undergo baseline brain imaging. A subset of stroke cases phenotypically identified as not “first ever stroke,” as recurrent and were derived from this data and are included in the current study.

REGARDS included all Black and White participants with available genotyping and informed consent. Individuals were excluded if they self-reported a history of stroke or lacked stroke follow-up information. Recurrent stroke cases are defined as participants with a primary incident ischemic stroke plus a secondary ischemic stroke during REGARDS follow-up.

For the SAHLSIS study to obtain data on non-fatal recurrent strokes, the National Hospital Discharge Registry was used. Sweden has a publicly financed healthcare system that offers healthcare to all citizens at a comparatively low cost, and all hospitals must report discharge diagnoses of all patients to this registry. The registry thus contains almost complete data (99%) on dates and codes for hospital discharge diagnoses and surgical procedures. All stroke diagnoses were confirmed by reviewing the corresponding medical record as described (Pedersen et al., 2016). When the medical record could not be found (in about 15 % of the cases), the event was still registered if it was the main diagnosis. Information on the cause of death was obtained from the Swedish Cause of Death Register, which is based on the International Classification of Diseases 10th Revision (ICD10). The medical records within 6 months before death were reviewed, both for participants who died in the hospital and for participants who died at home as described (Pedersen et al., 2016).

Recurrent stroke in VISP was defined as an incident stroke occurring after trial randomization and before the 2-year trial endpoint, which was previously described in great detail (Toole, 2002). In summary, recurrent stroke was diagnosed only with evidence of sudden onset of focal neurologic deficit lasting at least 24 h accompanied by an increased NIHSS score in a brain area that was previously normal. Determination of recurrent stroke status was done by a local neurologist and two external review committee neurologists.

For consistency, we defined recurrent stroke as an ischemic or hemorrhagic stroke that occurred after the “index” or qualifying ischemic stroke for study enrollment among the cohorts.

### 2.5 Statistical plan

#### 2.5.1 Genome-wide association studies

We performed African and European ancestry-stratified GWAS in the REGARDS and VISP cohorts due to the large proportions of these ancestries. Next, we meta-analyzed the REGARDS and VISP African ancestry GWAS summary statistics. We repeated this for European ancestry by adding the primarily European ASGC and SAHLSIS cohorts. Lastly, we performed a combined ancestry GWAS, including ASGC, REGARDS, SAHLSIS, and VISP. Each GWAS followed the same logistic regression model with Recurrent Stroke status as the response variable adjusting for age in years, sex, and the first 10 principal components. Because VISP was a clinical trial, we added the treatment arm to its respective models to account for possible confounding effects of vitamin supplementation.

#### 2.5.2 Meta-analysis

We utilized the “metafor” R package (Viechtbauer, 2010) to apply an inverse-variance weighted meta-analysis regression to the summary statistics from each ancestry type. The custom R script first performed the fixed effects method and calculated the *I*^2^-value. If the *I*^2^-value exceeded 50%, the script performed the same meta-analysis regression with the “REML” method for mixed-effect and the test parameter “knha.” The “knha” test is the Knapp-Hartung method, which applies an adjustment to the standard errors to account for uncertainty due to heterogeneity in the estimates. Due to the multiple hypothesis tests, we used a Bonferroni Correction set at two thresholds, genome-wide suggestive (*p* < 5e−6) and significant (*p* < 5e−8).

#### 2.5.3 Gene ontology enrichment and pathway analyses

We utilized the PANTHER classification system and software PANTHER18.0 (Thomas et al., 2022) to perform GO enrichment analysis for biological pathways in Homo sapiens using Fisher's Exact test and a False Discovery Rate (FDR) adjustment (Mi and Thomas, 2009). We also performed a PANTHER Pathways analysis using the same test and correction parameters (Mi et al., 2019).

#### 2.5.4 Integration of ischemic stroke polygenic risk scores

Much discussion surrounds the genetic risk of first-ever ischemic risk as a continued causal vector for the recurrent stroke phenotype. To help determine if the recurrent stroke meta-analyses reflect genetically determined ischemic stroke risk, we utilized the GIGAStroke consortium's polygenetic risk scores (PRS) for European ancestry (Mishra et al., 2022). We calculated scores within the entire VISP cohort (*n* = 2,100) and performed a Receiver Operating Characteristic (ROC) Curve analysis.

## 3 Results

### 3.1 Cohort demographics

All cohorts combined provide 4,420 stroke survivors for analysis with notable heterogeneity in recurrent stroke rates ranging from 8.6 to 29% (Table 1). Age differed similarly, with the SAHLSIS cohort having a median age of 58 compared to 76 years in the AUST cohort.

**Table 1:** Demographic characteristics by cohort.

**Cohort**	**ASGC (*n* = 580)**	**REGARDS (*n* = 926)**	**SAHLSIS (*n* = 931)**	**VISP (*n* = 1,983)**	**Total (*n* = 4,420)**
Recurrent stroke	168 (29%)	124 (13%)	146 (16%)	182 (8.7%)	620 (16.8%)
Age in years	76 (66, 82)	68 (62, 75)	58 (50, 64)	68 (60, 75)	66.9 (59.1, 73.6)
Male	262 (45%)	447 (48%)	596 (64%)	1,335 (64%)	660 (56.2%)
**Ancestry**					
African *N* (%)	-	560 (60%)	-	258 (13%)	818
European *N* (%)	580 (100%)	366 (40%)	931 (100%)	1,725 (87%)	3,602

### 3.2 GWAS meta-analyses

**Figure 1:**
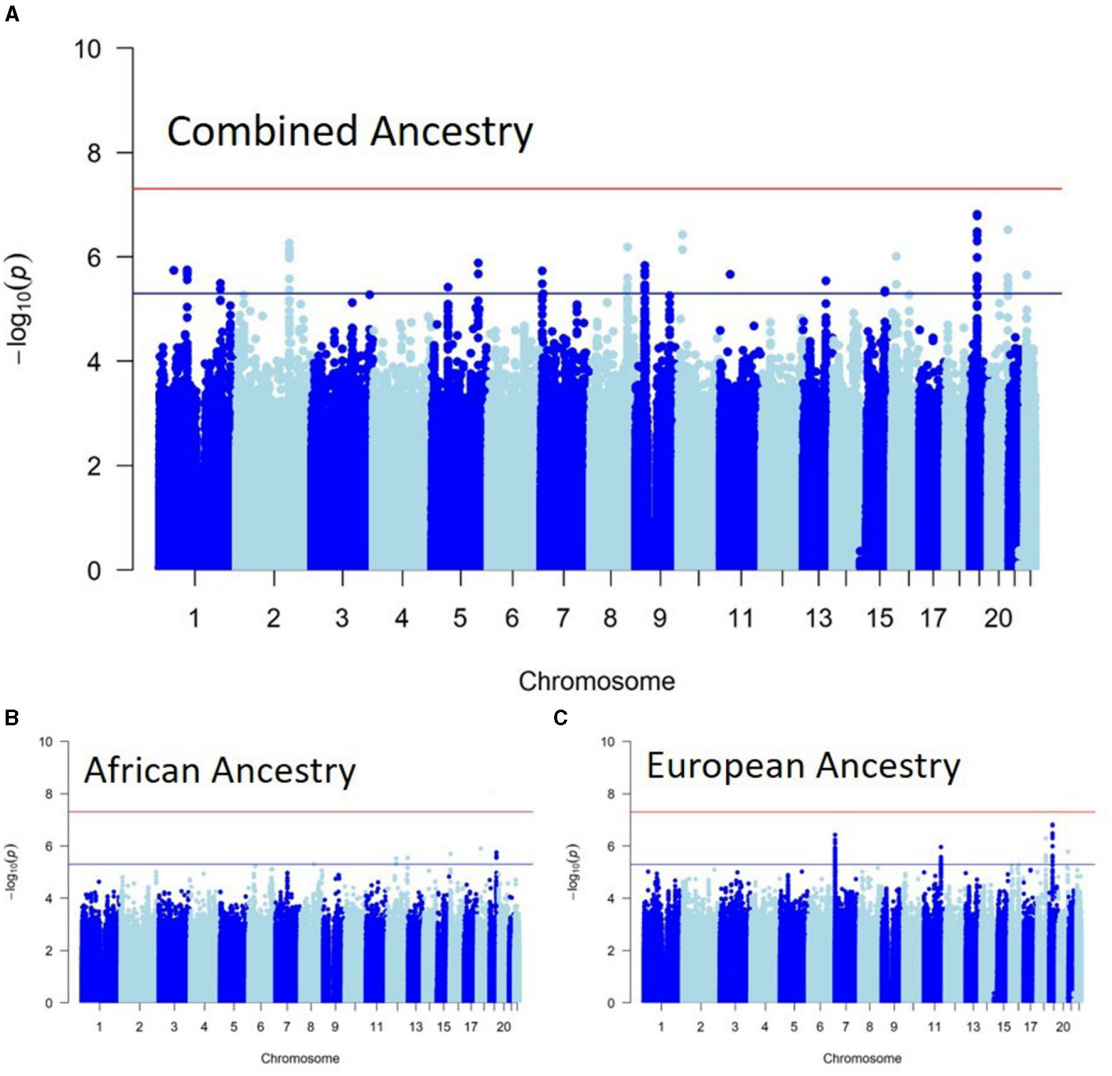
Ancestry-specific genome-wide association study (GWAS) meta-analyses. **(A)** Shows the genome-wide associations of recurrent stroke in individuals with African ancestry. **(B)** Shows the genome-wide associations of recurrent stroke in individuals with European ancestry, while **(C)** is the Combined ancestry GWAS meta-analysis.

**Figure 2:**
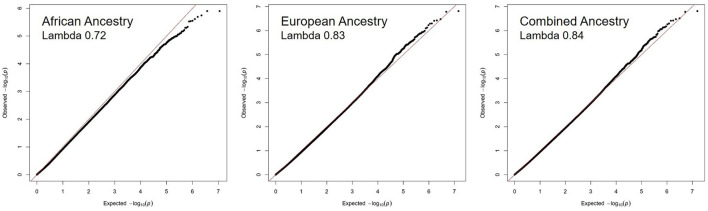
No evidence of genomic inflation of recurrent stroke GWAS meta-analysis across ancestries. The QQ plots for each ancestry reflect the observed –log10 (*p*-values) of each genetic variant vs. the –log10 (*p*-values) expected by chance due to multiple hypothesis testing. The lambda value quantifies the amount of inflation in the observed *p*-values, a value of 1.10 or less is acceptable.

Figure 1 shows the Manhattan plots of each Ancestry meta-analysis GWAS. The results of the ancestry-specific meta-analyses did not show any genomic inflation among the GWAS meta-analyses (Figure 2).

#### 3.2.1 African ancestry

The REGARDS and VISP African ancestry cohorts (*n* = 818) had 5,574,354 SNPs overlap. None of the genetic variants reached genome-wide significance. Nine reached suggestive significance (*p* < 5e−6), with some found within the *ADGRD1* (protein encoding), *PPL* (protein encoding), and *LINC01915* (long-coding RNA) genes (Supplementary material 2).

#### 3.2.2 European ancestry

The European ancestry meta-analysis consisted of the ASGC, REGARDS, SAHLSIS, and VISP cohorts (*n* = 3,602), with 6,140,939 SNPs overlapping at least two cohorts. Seventy-one SNPs reached suggestive associations (*p* < 5e−6) with a strong locus within the *SDK1* (protein encoding) gene (Supplementary material 2). There were other associations with the *RDX* (protein encoding) gene and *GAD3P* pseudogene.

#### 3.2.3 Combined ancestry

The combined ancestry meta-analysis consisted of all cohorts (*n* = 4,420) with African and European ancestries, which resulted in 7,948,212 overlapping SNPs. None of the SNPs reached genome-wide significance (*p* < 5e−8). However, 70 SNPs reached suggestive (*p* < 5e−6) associations (Supplementary material 2). Some of the suggestive associations are within the *CCDC3, CD59, CTXND1, DSCC1, GPC5, GSTT4, MAL2, MYH11, OPRL1, PELO-AS1, PPARGC1B, RN7SL396P*, and *SDK1* genes.

### 3.3 GO enrichment and PANTHER pathway analyses

We selected all of the genes with intron genetic variants from the combined ancestry meta-analysis with *P*-values < 5e−6, which were *CCDC3, CD59, CTXND1, DSCC1, GPC5, GSTT4, MAL2, MYH11, OPRL1, PELO-AS1, PPARGC1B, RN7SL396P*, and *SDK1*. Both the GO Enrichment and PANTHER Pathway analyses failed to show statistically significant associations after FDR correction.

### 3.4 Comparison of recurrent stroke meta-analysis with GIGAStroke

**Figure 3:**
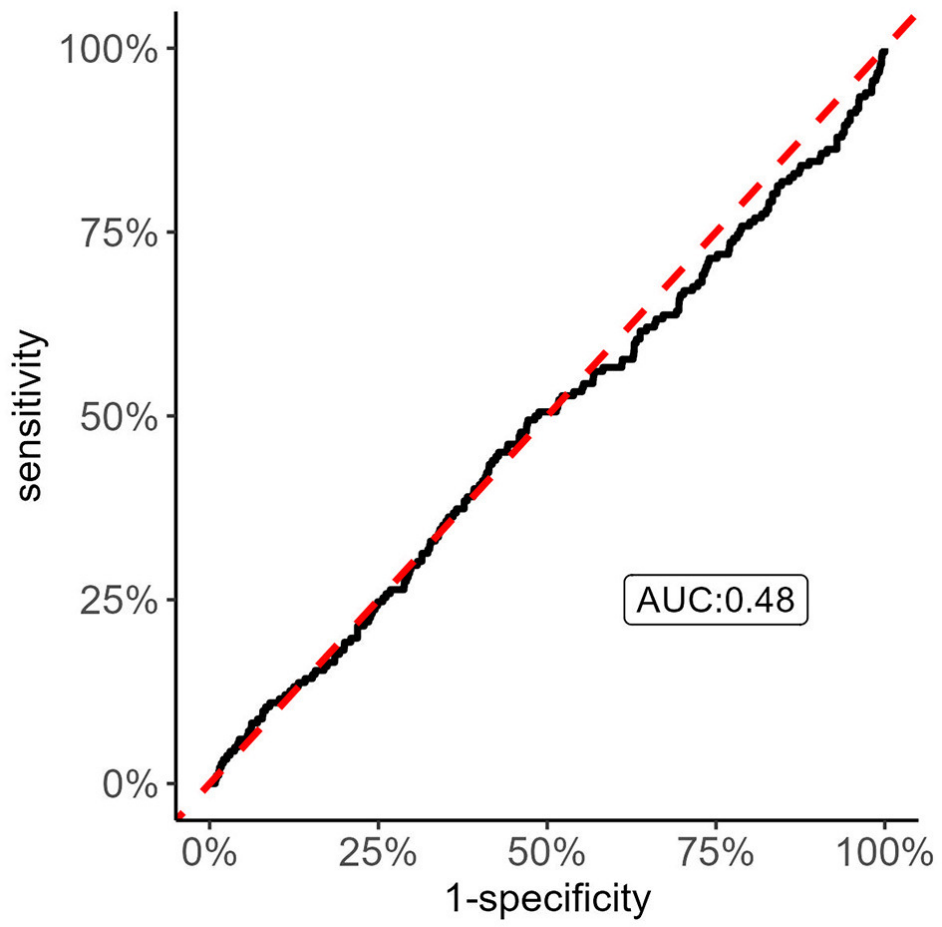
Receiver Operating Characteristic (ROC) curve analysis of recurrent stroke status with GIGASTROKE consortium's polygenic risk scores (PRS) of incident ischemic stroke. The figure shows that the incident ischemic stroke PRS do no better than chance in discriminating recurrent stroke status. Thus, there is the possibility that the genetic risk of recurrent stroke has distinct drivers compared to incident ischemic stroke.

We applied an ROC analysis on the calculated ischemic stroke PRS from the GIGAStroke consortium which revealed an area under the curve (AUC) of 0.48; Figure 3, suggesting that knowing the genetic risk of first-ever ischemic risk predicts recurrent stroke no better than chance (AUC = 0.5). Because of this result, we investigated if the PRS score was significantly associated with recurrent stroke status as a continuous variable and as quartiles via a Cox proportional hazards model. We set a significant level to *p* < 0.05. To avoid Simpson's paradox (Wagner, 1982), we added age, sex, and principal components 1–10 as covariates. Neither the continuous form nor the quartile one showed significant associations with recurrent stroke status. To note, there was a 97% overlap between the genetic variants within the GIGAStroke PRS and the VISP genotyped data.

Next, we investigated if any of the 60 genome-wide significant variants from GIGAStroke (Mishra et al., 2022), in their Supplementary Table 4A, were significant in our recurrent stroke meta-analysis results. None of the variants associated with stroke risk in GIGAStroke were associated with recurrent stroke in our study after Bonferroni correction (0.05/60).

**Figure 4:**
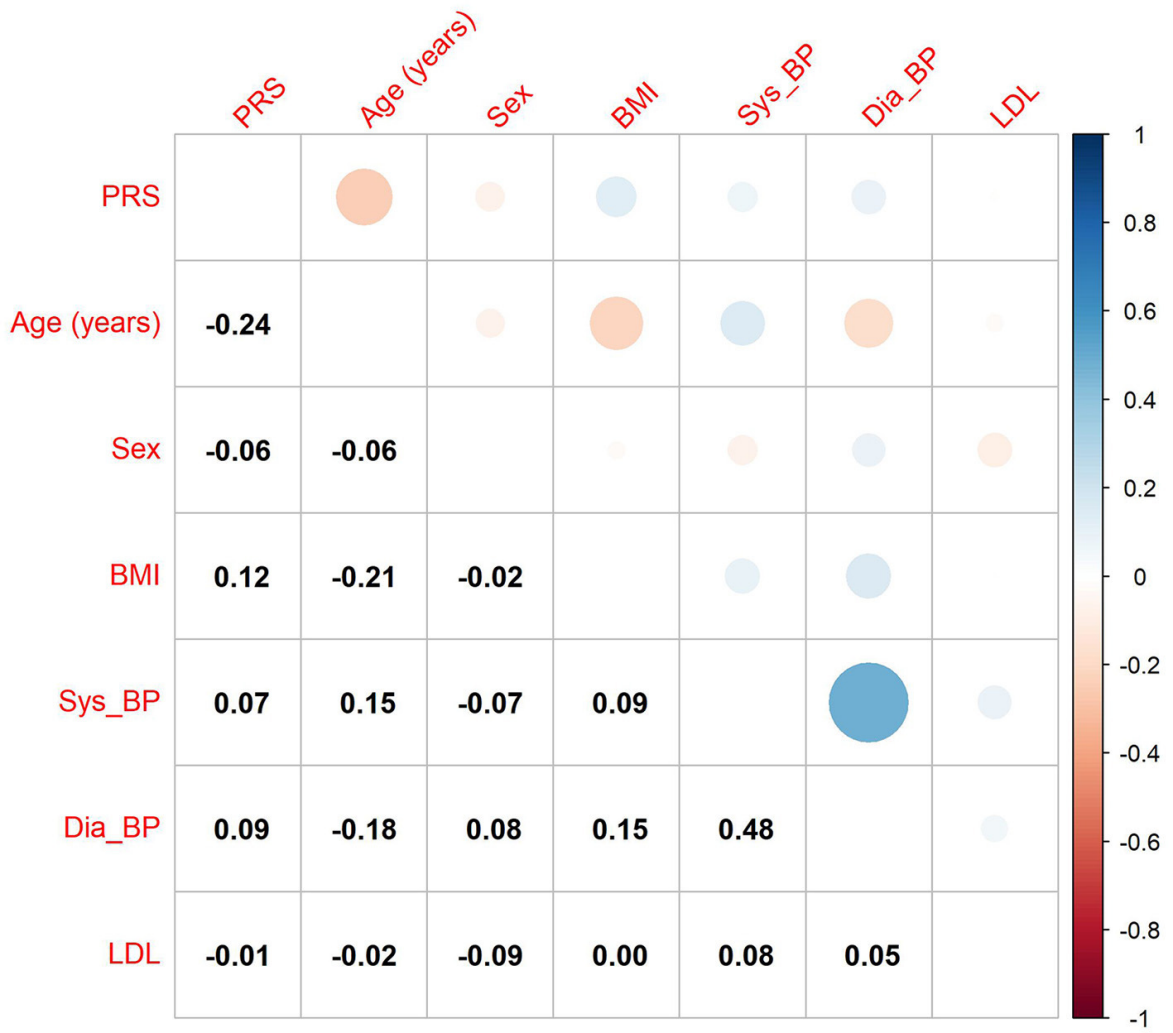
Correlation of GIGAStroke stroke polygenic risk score (PRS) with stroke risk factors in the VISP cohort. The black numbers show Pearson's correlation coefficient among all pairs. The size of the circles and their color reflect the direction and strength of the correlation coefficient, as seen on the upper triangle of the figure. PRS is the polygenic risk score for incident stroke, BMI is the body mass index value, and Sys_BP and Dia_BP are systolic and diastolic blood pressure.

Lastly, we investigated the correlation between the GIGAStroke PRS and known stroke risk factors within the VISP cohort (Figure 4). The PRS was most strongly associated with age in years (–R = 0.24; *p* < 0.001) followed by BMI (*R* = 0.12; *p* < 0.001).

## 4 Discussion

Stroke remains a leading cause of death and long-term disability worldwide. Strikingly, recurrent strokes are seven times more fatal, with high rates of disability after survival (Jong et al., 2004). GWAS offers a strong approach to discovering biological insights associated with recurrent stroke disease. Plus, performing ancestry-specific and combined GWAS meta-analyses provides a window into the genetic determinants of the recurrent stroke risk disparities between African and European ancestral groups. This study builds on prior work that investigated candidate gene associations with recurrent stroke (Fernández-Cadenas et al., 2017).

**Figure 5:**
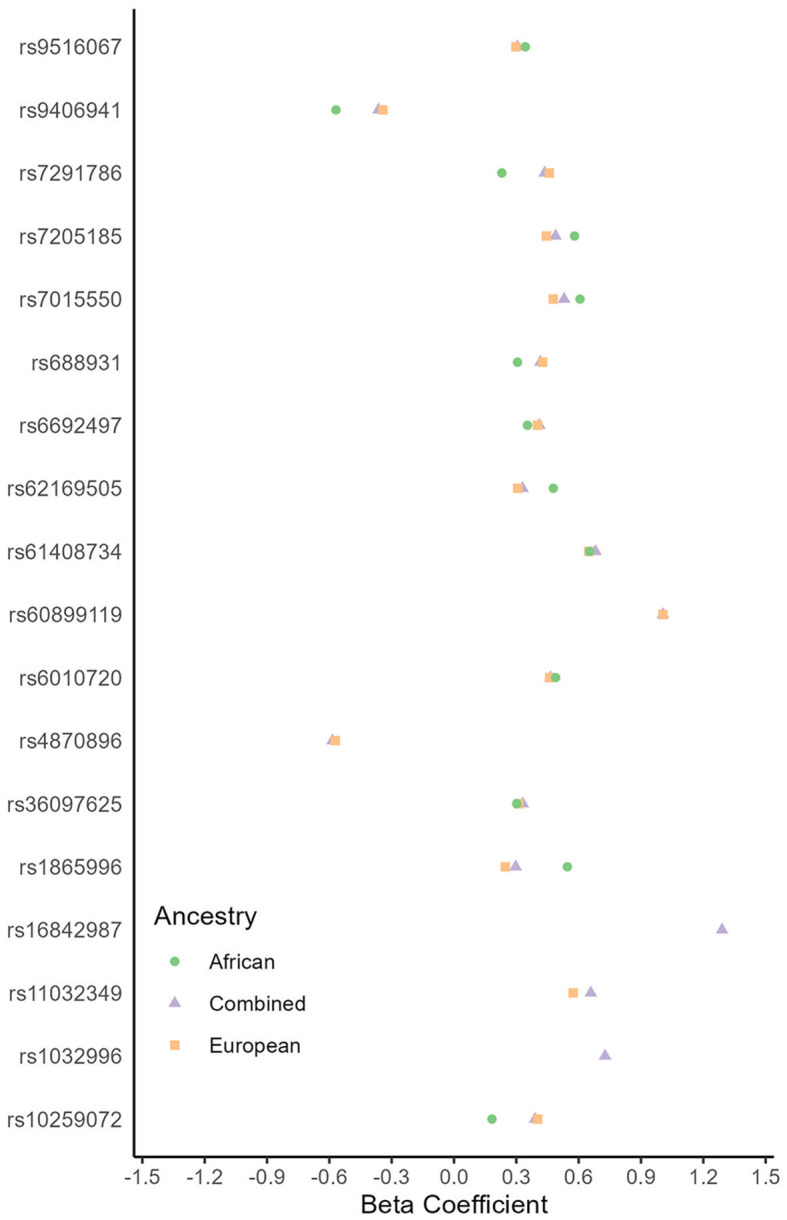
Genetic loci associated with recurrent stroke (*p* < 5e−6) by ancestry. Multiple genetic loci had estimates in the same direction (<0 or >0) between African and European ancestry genome-wide association studies. These genetic loci are consistent across ancestries.

We did not observe any genome-wide significant associations with recurrent stroke in any ancestry meta-analysis. However, we found 18 suggestive (*p* < 5e−6) genome-wide associated loci consistent among the African, European, and Combined ancestry meta-analyses (Supplementary material 2; Figure 5). Some appear related to stroke risk factors. For example, the *PPARGC1B* gene (rs61408734-T locus) is down-regulated in people with type 2 Diabetes Mellitus as well as in pre-diabetes (Patti et al., 2003; Ling et al., 2004). Additionally, the rs36097625-C locus within the *CCDC3* gene suggests an indirect link with tumor necrosis factor-alpha (TNF-alpha; Azad et al., 2014), which is a potential marker for stroke and its recovery (Xue et al., 2022). *CCDC3* plays an inhibitory role on TNF-alpha pro-inflammatory responses in endothelial cells, including vascular tissue (Azad et al., 2014). Similarly, *OPRL1* produces a G protein receptor known as the ORL1, N/OFQ receptor. This receptor receives the endogenous neuropeptide nociceptin which directly causes vasodilation in blood vessels. There is a proposed biological mechanism for cerebral hypoxia, wherein this receptor's down-regulation leads to vasoconstriction and the slowing of cerebrovascular blood flow (Armstead, 2011). Conversely, the *MYH11* gene (rs7205185-G) produces a smooth muscle myosin protein, which has been associated with aortic aneurysm/dissections (Zhu et al., 2006) with a theoretical mechanism of action being hyperplasia leading to an occlusive vascular pathology. The *DSCC1* gene also has associations with ascending aortic size and dispensability (Benjamins et al., 2022). Lastly, the *SDK1* gene has particular interest because of its link with hypertension in two prior GWAS in Nigerians (Tayo et al., 2009) and Japanese (Oguri et al., 2010) populations.

Other loci appear to be related to the central nervous system. Interestingly, the same rs7205185-G locus above is also found in the *NDE1* gene, because this gene and *MYH11* overlap the same section of chromosome 16, but on opposite strands. *NDE1* function is essential for the development of the cerebral cortex and may regulate production of neurons (Bakircioglu et al., 2011). The *CD59* gene (rs11032349-T locus) is a potent inhibitor of the complement membrane attack complex (MAC) with known expression in human central nervous tissue (Farkas et al., 2002). The *CD59* gene is slightly upregulated in neurons and glial cells in neuro-degenerative diseases associated with inflammation like Alzheimer's disease (McGeer et al., 1991).

The GO Enrichment and PANTHER Pathway analyses failed to show any biological process associations suggesting that this study has power limitations. Because of this and the desire to determine how much genetic risk of ischemic stroke can explain recurrent stroke risk, we implemented the GIGAStroke Consortium's PRS for European ancestry in the VISP cohort. The European PRS was developed from a primarily European cohort, but the cohort also had a notable proportion of individuals with African ancestry (Mishra et al., 2022). This is similar to the VISP cohort. We found that the PRS did no better than chance when discriminating recurrent stroke cases vs. control. By investigating stroke survivors, we are conditioning on the fact that they had a stroke and are already genetically “at-risk” for stroke.

Because the GIGAStroke consortium's PRS values correlated negatively with age and positively with BMI (Figure 4) in the VISP cohort, there is a measure of validity. Individuals with higher genetic risk of stroke are more likely have a stroke at a younger age, and BMI is a well-known stroke risk-factor. This raises the possibility that genetically determined recurrent stroke risk has some distinct signatures from incident stroke risk. Therefore, additional recurrent stroke GWAS must address the question: are there independent genetic drivers of recurrent stroke compared to incident stroke? This is an important question to help direct the field to new therapeutics for mechanism-specific secondary stroke prevention.

It is important to note that our study with 620/4,420 recurrent stroke cases among all ancestries is likely an underpowered GWAS for such a complex and multi-factorial disease. This may have contributed to the lack of genome-wide significant associations. Furthermore, our genome-wide suggestive (*p* < 5e−6) associations have higher a probability of false positive associations due to the large number of hypothesis tests. On the other hand, the majority of the suggestive genetic loci have known biological mechanisms affecting vascular contractility and stenosis as well as neuron development or response. The addition of strong biological relevance provides greater confidence in these suggestive genetic loci for further investigation.

## 5 Conclusion

In conclusion, we discovered several suggestive genetic loci associated with recurrent stroke from a population of African and European ancestries. Even though our recurrent stroke GWAS meta-analyses failed to find genome-wide significant associations, we observed multiple suggestive loci with high biological relevance. The application of incident ischemic stroke PRS to the VISP cohort revealed poor ability to discriminate between recurrent stroke status. We believe that this suggests that recurrent stroke genetic drivers are at least in part distinct from incident ischemic stroke.

## Data availability statement

The datasets presented in this study can be found in online repositories. The names of the repository/repositories and accession number(s) can be found in the article/Supplementary material.

## Ethics statement

The studies involving humans were approved by Wake Forest University School of Medicine University of North Carolina at Chapel Hill School of Medicine. The studies were conducted in accordance with the local legislation and institutional requirements. Written informed consent for participation was not required from the participants or the participants' legal guardians/next of kin in accordance with the national legislation and institutional requirements.

## Author contributions

CA: Formal analysis, Methodology, Writing – original draft. NA: Formal analysis, Methodology, Writing – review & editing. NS: Writing – review & editing. CB: Writing – review & editing. DP: Writing – review & editing. AL: Writing – review & editing. AP: Data curation, Writing – review & editing. TS: Data curation, Funding acquisition, Writing – review & editing. CJ: Data curation, Funding acquisition, Writing – review & editing. JM: Data curation, Funding acquisition, Writing – review & editing. F-CH: Methodology, Supervision, Writing – review & editing. KK: Methodology, Supervision, Writing – review & editing. MI: Data curation, Writing – review & editing. MS: Funding acquisition, Methodology, Writing – review & editing. BW: Data curation, Funding acquisition, Methodology, Supervision, Writing – review & editing.
