# Supplementary material for: Diversity in genetic risk of recurrent stroke: a genome-wide association study meta-analysis
Source: Front Stroke. 2024 Feb 21;3:1338636. doi: 10.3389/fstro.2024.1338636 (PMC12802773; doi:10.3389/fstro.2024.1338636)
Supplement: Supplementary file 1 [file Table_1.DOCX]

| marker.ID | estimate | std.error | odds_ratio | conf.low | conf.high | p.value | method | ancestry | effect_allele | gene | biotype |
| --- | --- | --- | --- | --- | --- | --- | --- | --- | --- | --- | --- |
| rs1032996 | | | | | | | | | | | |
| chr1:47578015:C:A | 0.73 | 0.15 | 2.07 | 1.53 | 2.79 | 1.85 × 10^-6 | FE | Combined | C |  | enhancer |
| rs56250415 | | | | | | | | | | | |
| chr1:90679792:CAA:C | 0.41 | 0.09 | 1.51 | 1.27 | 1.79 | 2.09 × 10^-6 | FE | Combined | C |  |  |
| chr1:90679792:CAA:C | 0.40 | 0.09 | 1.49 | 1.23 | 1.79 | 2.78 × 10^-5 | FE | European | C |  |  |
| chr1:90679792:CAA:C | 0.40 | 0.23 | 1.50 | 0.96 | 2.33 | 7.35 × 10^-2 | FE | African | C |  |  |
| rs796567398 | | | | | | | | | | | |
| chr1:90679792:CAA:C | 0.41 | 0.09 | 1.51 | 1.27 | 1.79 | 2.09 × 10^-6 | FE | Combined | C |  |  |
| chr1:90679792:CAA:C | 0.40 | 0.09 | 1.49 | 1.23 | 1.79 | 2.78 × 10^-5 | FE | European | C |  |  |
| chr1:90679792:CAA:C | 0.40 | 0.23 | 1.50 | 0.96 | 2.33 | 7.35 × 10^-2 | FE | African | C |  |  |
| rs55985644 | | | | | | | | | | | |
| chr1:90679890:G:C | 0.41 | 0.09 | 1.51 | 1.27 | 1.79 | 2.15 × 10^-6 | FE | Combined | C |  |  |
| chr1:90679890:G:C | 0.40 | 0.09 | 1.49 | 1.24 | 1.80 | 2.14 × 10^-5 | FE | European | C |  |  |
| chr1:90679890:G:C | 0.37 | 0.23 | 1.45 | 0.93 | 2.26 | 1.03 × 10^-1 | FE | African | C |  |  |
| rs6666331 | | | | | | | | | | | |
| chr1:90680389:C:T | 0.41 | 0.09 | 1.51 | 1.27 | 1.79 | 1.90 × 10^-6 | FE | Combined | T |  |  |
| chr1:90680389:C:T | 0.40 | 0.09 | 1.49 | 1.24 | 1.79 | 2.55 × 10^-5 | FE | European | T |  |  |
| chr1:90680389:C:T | 0.40 | 0.23 | 1.50 | 0.96 | 2.33 | 7.35 × 10^-2 | FE | African | T |  |  |
| rs4658167 | | | | | | | | | | | |
| chr1:90681169:A:G | 0.41 | 0.09 | 1.51 | 1.27 | 1.79 | 2.02 × 10^-6 | FE | Combined | G |  |  |
| chr1:90681169:A:G | 0.40 | 0.09 | 1.49 | 1.24 | 1.79 | 2.69 × 10^-5 | FE | European | G |  |  |
| chr1:90681169:A:G | 0.40 | 0.23 | 1.50 | 0.96 | 2.33 | 7.35 × 10^-2 | FE | African | G |  |  |
| rs12096247 | | | | | | | | | | | |
| chr1:90682880:A:G | 0.40 | 0.09 | 1.50 | 1.27 | 1.77 | 2.78 × 10^-6 | FE | Combined | G |  |  |
| chr1:90682880:A:G | 0.40 | 0.09 | 1.49 | 1.24 | 1.80 | 2.05 × 10^-5 | FE | European | G |  |  |
| chr1:90682880:A:G | 0.31 | 0.22 | 1.37 | 0.88 | 2.11 | 1.59 × 10^-1 | FE | African | G |  |  |
| rs12401375 | | | | | | | | | | | |
| chr1:90683976:A:G | 0.41 | 0.09 | 1.51 | 1.27 | 1.78 | 1.85 × 10^-6 | FE | Combined | G |  |  |
| chr1:90683976:A:G | 0.40 | 0.09 | 1.49 | 1.24 | 1.80 | 2.05 × 10^-5 | FE | European | G |  |  |
| chr1:90683976:A:G | 0.35 | 0.22 | 1.42 | 0.93 | 2.19 | 1.06 × 10^-1 | FE | African | G |  |  |
| rs6679774 | | | | | | | | | | | |
| chr1:90684037:C:A | 0.41 | 0.09 | 1.50 | 1.27 | 1.78 | 2.27 × 10^-6 | FE | Combined | A |  |  |
| chr1:90684037:C:A | 0.40 | 0.09 | 1.49 | 1.24 | 1.80 | 2.22 × 10^-5 | FE | European | A |  |  |
| chr1:90684037:C:A | 0.34 | 0.22 | 1.40 | 0.92 | 2.15 | 1.20 × 10^-1 | FE | African | A |  |  |
| rs6692497 | | | | | | | | | | | |
| chr1:90684372:A:G | 0.41 | 0.09 | 1.51 | 1.27 | 1.79 | 1.81 × 10^-6 | FE | Combined | G |  |  |
| chr1:90684372:A:G | 0.40 | 0.09 | 1.50 | 1.24 | 1.80 | 2.01 × 10^-5 | FE | European | G |  |  |
| chr1:90684372:A:G | 0.35 | 0.22 | 1.42 | 0.93 | 2.19 | 1.06 × 10^-1 | FE | African | G |  |  |
| rs963563 | | | | | | | | | | | |
| chr1:198365681:G:T | 1.29 | 0.28 | 3.63 | 2.10 | 6.30 | 4.20 × 10^-6 | FE | Combined | T |  |  |
| rs58760485 | | | | | | | | | | | |
| chr1:198377078:A:G | 1.29 | 0.28 | 3.63 | 2.10 | 6.30 | 4.20 × 10^-6 | FE | Combined | G |  |  |
| rs16842987 | | | | | | | | | | | |
| chr1:198402017:G:A | 1.29 | 0.28 | 3.63 | 2.11 | 6.25 | 3.24 × 10^-6 | FE | Combined | A |  |  |
| rs62169505 | | | | | | | | | | | |
| chr2:170686866:A:G | 0.33 | 0.07 | 1.39 | 1.22 | 1.58 | 5.51 × 10^-7 | FE | Combined | G |  | lncRNA, misc_RNA |
| chr2:170686866:A:G | 0.31 | 0.07 | 1.36 | 1.18 | 1.56 | 2.04 × 10^-5 | FE | European | G |  | lncRNA, misc_RNA |
| chr2:170686866:A:G | 0.48 | 0.17 | 1.61 | 1.16 | 2.25 | 4.64 × 10^-3 | FE | African | G |  | lncRNA, misc_RNA |
| rs61162914 | | | | | | | | | | | |
| chr2:170687270:G:A | 0.31 | 0.07 | 1.36 | 1.20 | 1.55 | 2.70 × 10^-6 | FE | Combined | A |  | lncRNA, misc_RNA |
| chr2:170687270:G:A | 0.31 | 0.07 | 1.36 | 1.18 | 1.57 | 2.08 × 10^-5 | FE | European | A |  | lncRNA, misc_RNA |
| chr2:170687270:G:A | 0.30 | 0.29 | 1.35 | 0.77 | 2.37 | 4.87 × 10^-1 | REML | African | A |  | lncRNA, misc_RNA |
| rs200413762 | | | | | | | | | | | |
| chr2:170688104:T:TG | 0.33 | 0.07 | 1.39 | 1.22 | 1.58 | 7.63 × 10^-7 | FE | Combined | TG |  | lncRNA, misc_RNA |
| chr2:170688104:T:TG | 0.30 | 0.07 | 1.35 | 1.17 | 1.56 | 3.10 × 10^-5 | FE | European | TG |  | lncRNA, misc_RNA |
| chr2:170688104:T:TG | 0.49 | 0.17 | 1.63 | 1.17 | 2.27 | 3.60 × 10^-3 | FE | African | TG |  | lncRNA, misc_RNA |
| rs75187838 | | | | | | | | | | | |
| chr2:170688199:A:G | 0.33 | 0.07 | 1.38 | 1.22 | 1.58 | 8.90 × 10^-7 | FE | Combined | G |  | lncRNA, misc_RNA |
| chr2:170688199:A:G | 0.30 | 0.07 | 1.35 | 1.17 | 1.56 | 3.57 × 10^-5 | FE | European | G |  | lncRNA, misc_RNA |
| chr2:170688199:A:G | 0.49 | 0.17 | 1.63 | 1.17 | 2.27 | 3.69 × 10^-3 | FE | African | G |  | lncRNA, misc_RNA |
| rs79747073 | | | | | | | | | | | |
| chr2:170688206:G:A | 0.32 | 0.07 | 1.38 | 1.21 | 1.57 | 1.06 × 10^-6 | FE | Combined | A |  | lncRNA, misc_RNA |
| chr2:170688206:G:A | 0.30 | 0.07 | 1.34 | 1.17 | 1.55 | 4.17 × 10^-5 | FE | European | A |  | lncRNA, misc_RNA |
| chr2:170688206:G:A | 0.49 | 0.17 | 1.63 | 1.17 | 2.27 | 3.69 × 10^-3 | FE | African | A |  | lncRNA, misc_RNA |
| rs77271852 | | | | | | | | | | | |
| chr2:170688215:A:G | 0.33 | 0.07 | 1.39 | 1.22 | 1.58 | 8.76 × 10^-7 | FE | Combined | G |  | lncRNA, misc_RNA |
| chr2:170688215:A:G | 0.30 | 0.07 | 1.35 | 1.17 | 1.56 | 3.52 × 10^-5 | FE | European | G |  | lncRNA, misc_RNA |
| chr2:170688215:A:G | 0.49 | 0.17 | 1.63 | 1.17 | 2.27 | 3.69 × 10^-3 | FE | African | G |  | lncRNA, misc_RNA |
| rs78701078 | | | | | | | | | | | |
| chr2:170688221:T:C | 0.33 | 0.07 | 1.39 | 1.22 | 1.58 | 8.76 × 10^-7 | FE | Combined | C |  | lncRNA, misc_RNA |
| chr2:170688221:T:C | 0.30 | 0.07 | 1.35 | 1.17 | 1.56 | 3.52 × 10^-5 | FE | European | C |  | lncRNA, misc_RNA |
| chr2:170688221:T:C | 0.49 | 0.17 | 1.63 | 1.17 | 2.27 | 3.69 × 10^-3 | FE | African | C |  | lncRNA, misc_RNA |
| rs6433230 | | | | | | | | | | | |
| chr2:170688960:C:A | 0.33 | 0.07 | 1.39 | 1.22 | 1.58 | 7.26 × 10^-7 | FE | Combined | A |  | lncRNA, misc_RNA |
| chr2:170688960:C:A | 0.30 | 0.07 | 1.35 | 1.17 | 1.56 | 3.09 × 10^-5 | FE | European | A |  | lncRNA, misc_RNA |
| chr2:170688960:C:A | 0.50 | 0.17 | 1.65 | 1.18 | 2.31 | 3.57 × 10^-3 | FE | African | A |  | lncRNA, misc_RNA |
| rs6433232 | | | | | | | | | | | |
| chr2:170689703:G:A | 0.31 | 0.07 | 1.36 | 1.19 | 1.55 | 4.36 × 10^-6 | FE | Combined | A |  | lncRNA, misc_RNA, enhancer |
| chr2:170689703:G:A | 0.28 | 0.07 | 1.33 | 1.15 | 1.53 | 1.18 × 10^-4 | FE | European | A |  | lncRNA, misc_RNA, enhancer |
| chr2:170689703:G:A | 0.45 | 0.17 | 1.56 | 1.12 | 2.19 | 9.04 × 10^-3 | FE | African | A |  | lncRNA, misc_RNA, enhancer |
| rs688931 | | | | | | | | | | | |
| chr5:52757074:T:G | 0.42 | 0.09 | 1.52 | 1.27 | 1.81 | 3.91 × 10^-6 | FE | Combined | G | PELO-AS1 | lncRNA |
| chr5:52757074:T:G | 0.43 | 0.10 | 1.53 | 1.26 | 1.86 | 1.33 × 10^-5 | FE | European | G | PELO-AS1 | lncRNA |
| chr5:52757074:T:G | 0.31 | 0.23 | 1.36 | 0.86 | 2.14 | 1.88 × 10^-1 | FE | African | G | PELO-AS1 | lncRNA |
| rs56938831 | | | | | | | | | | | |
| chr5:149848298:A:T | 0.67 | 0.14 | 1.95 | 1.48 | 2.57 | 2.17 × 10^-6 | FE | Combined | T | PPARGC1B | protein_coding, retained_intron |
| chr5:149848298:A:T | 0.65 | 0.21 | 1.92 | 1.27 | 2.88 | 1.84 × 10^-3 | FE | European | T | PPARGC1B | protein_coding, retained_intron |
| chr5:149848298:A:T | 0.62 | 0.20 | 1.87 | 1.27 | 2.74 | 1.49 × 10^-3 | FE | African | T | PPARGC1B | protein_coding, retained_intron |
| rs61408734 | | | | | | | | | | | |
| chr5:149849522:C:T | 0.68 | 0.14 | 1.98 | 1.50 | 2.60 | 1.34 × 10^-6 | FE | Combined | T | PPARGC1B | protein_coding, retained_intron |
| chr5:149849522:C:T | 0.65 | 0.21 | 1.91 | 1.27 | 2.88 | 1.85 × 10^-3 | FE | European | T | PPARGC1B | protein_coding, retained_intron |
| chr5:149849522:C:T | 0.65 | 0.20 | 1.92 | 1.31 | 2.83 | 9.30 × 10^-4 | FE | African | T | PPARGC1B | protein_coding, retained_intron |
| rs10499336 | | | | | | | | | | | |
| chr7:3482101:C:G | 0.31 | 0.15 | 1.36 | 1.02 | 1.83 | 1.07 × 10^-1 | REML | Combined | G | SDK1 | protein_coding |
| chr7:3482101:C:G | 0.42 | 0.08 | 1.52 | 1.29 | 1.79 | 3.79 × 10^-7 | FE | European | G | SDK1 | protein_coding |
| chr7:3482101:C:G | -0.51 | 0.25 | 0.60 | 0.37 | 0.98 | 4.03 × 10^-2 | FE | African | G | SDK1 | protein_coding |
| rs57733415 | | | | | | | | | | | |
| chr7:3485022:A:G | 0.22 | 0.17 | 1.25 | 0.90 | 1.73 | 2.55 × 10^-1 | REML | Combined | G | SDK1 | protein_coding |
| chr7:3485022:A:G | 0.36 | 0.08 | 1.44 | 1.23 | 1.68 | 3.86 × 10^-6 | FE | European | G | SDK1 | protein_coding |
| chr7:3485022:A:G | -0.46 | 0.20 | 0.63 | 0.42 | 0.94 | 2.21 × 10^-2 | FE | African | G | SDK1 | protein_coding |
| rs17133420 | | | | | | | | | | | |
| chr7:3486560:C:A | 0.22 | 0.16 | 1.25 | 0.91 | 1.72 | 2.41 × 10^-1 | REML | Combined | A | SDK1 | protein_coding |
| chr7:3486560:C:A | 0.36 | 0.08 | 1.44 | 1.23 | 1.68 | 3.63 × 10^-6 | FE | European | A | SDK1 | protein_coding |
| chr7:3486560:C:A | -0.48 | 0.21 | 0.62 | 0.41 | 0.92 | 1.85 × 10^-2 | FE | African | A | SDK1 | protein_coding |
| rs17133432 | | | | | | | | | | | |
| chr7:3490619:G:C | 0.25 | 0.16 | 1.29 | 0.93 | 1.77 | 2.01 × 10^-1 | REML | Combined | C | SDK1 | protein_coding |
| chr7:3490619:G:C | 0.36 | 0.08 | 1.44 | 1.23 | 1.68 | 4.62 × 10^-6 | FE | European | C | SDK1 | protein_coding |
| chr7:3490619:G:C | -0.59 | 0.28 | 0.55 | 0.32 | 0.96 | 3.44 × 10^-2 | FE | African | C | SDK1 | protein_coding |
| rs2141819 | | | | | | | | | | | |
| chr7:3491514:G:A | 0.22 | 0.18 | 1.25 | 0.88 | 1.76 | 2.78 × 10^-1 | REML | Combined | A | SDK1 | protein_coding |
| chr7:3491514:G:A | 0.35 | 0.07 | 1.42 | 1.22 | 1.64 | 2.65 × 10^-6 | FE | European | A | SDK1 | protein_coding |
| chr7:3491514:G:A | -0.39 | 0.16 | 0.68 | 0.49 | 0.94 | 1.83 × 10^-2 | FE | African | A | SDK1 | protein_coding |
| rs2141821 | | | | | | | | | | | |
| chr7:3491643:C:T | 0.22 | 0.18 | 1.24 | 0.88 | 1.76 | 2.87 × 10^-1 | REML | Combined | T | SDK1 | protein_coding |
| chr7:3491643:C:T | 0.34 | 0.07 | 1.41 | 1.22 | 1.63 | 3.64 × 10^-6 | FE | European | T | SDK1 | protein_coding |
| chr7:3491643:C:T | -0.39 | 0.16 | 0.67 | 0.49 | 0.93 | 1.70 × 10^-2 | FE | African | T | SDK1 | protein_coding |
| rs2177169 | | | | | | | | | | | |
| chr7:3492263:A:G | 0.21 | 0.18 | 1.24 | 0.88 | 1.75 | 2.91 × 10^-1 | REML | Combined | G | SDK1 | protein_coding |
| chr7:3492263:A:G | 0.34 | 0.07 | 1.40 | 1.21 | 1.62 | 4.56 × 10^-6 | FE | European | G | SDK1 | protein_coding |
| chr7:3492263:A:G | -0.39 | 0.16 | 0.68 | 0.49 | 0.94 | 1.91 × 10^-2 | FE | African | G | SDK1 | protein_coding |
| rs55643551 | | | | | | | | | | | |
| chr7:3492338:G:C | 0.20 | 0.19 | 1.22 | 0.85 | 1.77 | 3.45 × 10^-1 | REML | Combined | C | SDK1 | protein_coding |
| chr7:3492338:G:C | 0.34 | 0.07 | 1.40 | 1.21 | 1.62 | 4.48 × 10^-6 | FE | European | C | SDK1 | protein_coding |
| chr7:3492338:G:C | -0.44 | 0.17 | 0.64 | 0.46 | 0.89 | 8.25 × 10^-3 | FE | African | C | SDK1 | protein_coding |
| rs2140115 | | | | | | | | | | | |
| chr7:3492363:A:G | 0.21 | 0.18 | 1.24 | 0.88 | 1.75 | 2.92 × 10^-1 | REML | Combined | G | SDK1 | protein_coding |
| chr7:3492363:A:G | 0.34 | 0.07 | 1.40 | 1.21 | 1.62 | 4.82 × 10^-6 | FE | European | G | SDK1 | protein_coding |
| chr7:3492363:A:G | -0.39 | 0.16 | 0.68 | 0.49 | 0.94 | 1.92 × 10^-2 | FE | African | G | SDK1 | protein_coding |
| rs11531491 | | | | | | | | | | | |
| chr7:3493024:C:T | 0.28 | 0.18 | 1.33 | 0.93 | 1.90 | 1.96 × 10^-1 | REML | Combined | T | SDK1 | protein_coding |
| chr7:3493024:C:T | 0.43 | 0.09 | 1.54 | 1.29 | 1.83 | 1.43 × 10^-6 | FE | European | T | SDK1 | protein_coding |
| chr7:3493024:C:T | -0.61 | 0.28 | 0.54 | 0.31 | 0.95 | 3.07 × 10^-2 | FE | African | T | SDK1 | protein_coding |
| rs17133439 | | | | | | | | | | | |
| chr7:3494209:G:C | 0.25 | 0.23 | 1.29 | 0.83 | 2.01 | 3.47 × 10^-1 | REML | Combined | C | SDK1 | protein_coding |
| chr7:3494209:G:C | 0.42 | 0.09 | 1.53 | 1.28 | 1.82 | 2.96 × 10^-6 | FE | European | C | SDK1 | protein_coding |
| chr7:3494209:G:C | -0.62 | 0.28 | 0.54 | 0.31 | 0.93 | 2.73 × 10^-2 | FE | African | C | SDK1 | protein_coding |
| rs17133445 | | | | | | | | | | | |
| chr7:3496095:G:A | 0.28 | 0.21 | 1.32 | 0.88 | 1.98 | 2.72 × 10^-1 | REML | Combined | A | SDK1 | protein_coding |
| chr7:3496095:G:A | 0.43 | 0.09 | 1.54 | 1.29 | 1.84 | 1.80 × 10^-6 | FE | European | A | SDK1 | protein_coding |
| chr7:3496095:G:A | -0.51 | 0.27 | 0.60 | 0.36 | 1.02 | 5.73 × 10^-2 | FE | African | A | SDK1 | protein_coding |
| rs16870870 | | | | | | | | | | | |
| chr7:3502569:G:A | 0.25 | 0.23 | 1.29 | 0.83 | 2.00 | 3.44 × 10^-1 | REML | Combined | A | SDK1 | protein_coding |
| chr7:3502569:G:A | 0.42 | 0.09 | 1.52 | 1.27 | 1.81 | 3.45 × 10^-6 | FE | European | A | SDK1 | protein_coding |
| chr7:3502569:G:A | -0.57 | 0.27 | 0.57 | 0.33 | 0.97 | 3.68 × 10^-2 | FE | African | A | SDK1 | protein_coding |
| rs7789656 | | | | | | | | | | | |
| chr7:3507288:A:C | 0.25 | 0.22 | 1.28 | 0.83 | 1.99 | 3.50 × 10^-1 | REML | Combined | C | SDK1 | protein_coding |
| chr7:3507288:A:C | 0.41 | 0.09 | 1.51 | 1.27 | 1.80 | 4.25 × 10^-6 | FE | European | C | SDK1 | protein_coding |
| chr7:3507288:A:C | -0.61 | 0.28 | 0.54 | 0.31 | 0.94 | 2.89 × 10^-2 | FE | African | C | SDK1 | protein_coding |
| rs1522505 | | | | | | | | | | | |
| chr7:3507574:T:G | 0.28 | 0.19 | 1.32 | 0.90 | 1.93 | 2.48 × 10^-1 | REML | Combined | G | SDK1 | protein_coding |
| chr7:3507574:T:G | 0.41 | 0.09 | 1.51 | 1.27 | 1.81 | 4.17 × 10^-6 | FE | European | G | SDK1 | protein_coding |
| chr7:3507574:T:G | -0.46 | 0.26 | 0.63 | 0.38 | 1.05 | 7.82 × 10^-2 | FE | African | G | SDK1 | protein_coding |
| rs77521796 | | | | | | | | | | | |
| chr7:3508580:G:C | 0.26 | 0.21 | 1.29 | 0.85 | 1.96 | 3.11 × 10^-1 | REML | Combined | C | SDK1 | protein_coding |
| chr7:3508580:G:C | 0.41 | 0.09 | 1.51 | 1.27 | 1.80 | 4.55 × 10^-6 | FE | European | C | SDK1 | protein_coding |
| chr7:3508580:G:C | -0.53 | 0.27 | 0.59 | 0.35 | 0.99 | 4.68 × 10^-2 | FE | African | C | SDK1 | protein_coding |
| rs112616734 | | | | | | | | | | | |
| chr7:3528128:C:T | 0.44 | 0.10 | 1.55 | 1.28 | 1.87 | 6.86 × 10^-6 | FE | Combined | T | SDK1 | protein_coding |
| chr7:3528128:C:T | 0.46 | 0.10 | 1.58 | 1.30 | 1.91 | 2.57 × 10^-6 | FE | European | T | SDK1 | protein_coding |
| rs12111969 | | | | | | | | | | | |
| chr7:3531657:G:A | 0.27 | 0.16 | 1.31 | 0.96 | 1.77 | 1.59 × 10^-1 | REML | Combined | A | SDK1 | protein_coding |
| chr7:3531657:G:A | 0.41 | 0.09 | 1.50 | 1.27 | 1.78 | 2.98 × 10^-6 | FE | European | A | SDK1 | protein_coding |
| chr7:3531657:G:A | -0.42 | 0.23 | 0.66 | 0.42 | 1.04 | 7.18 × 10^-2 | FE | African | A | SDK1 | protein_coding |
| rs9771445 | | | | | | | | | | | |
| chr7:3532130:A:G | 0.28 | 0.13 | 1.32 | 1.02 | 1.72 | 1.04 × 10^-1 | REML | Combined | G | SDK1 | protein_coding |
| chr7:3532130:A:G | 0.40 | 0.09 | 1.50 | 1.26 | 1.77 | 3.77 × 10^-6 | FE | European | G | SDK1 | protein_coding |
| chr7:3532130:A:G | -0.36 | 0.22 | 0.70 | 0.45 | 1.08 | 1.04 × 10^-1 | FE | African | G | SDK1 | protein_coding |
| rs7791215 | | | | | | | | | | | |
| chr7:3532323:A:G | 0.27 | 0.14 | 1.31 | 1.01 | 1.72 | 1.16 × 10^-1 | REML | Combined | G | SDK1 | protein_coding |
| chr7:3532323:A:G | 0.40 | 0.09 | 1.49 | 1.26 | 1.77 | 3.91 × 10^-6 | FE | European | G | SDK1 | protein_coding |
| chr7:3532323:A:G | -0.38 | 0.22 | 0.69 | 0.45 | 1.05 | 8.57 × 10^-2 | FE | African | G | SDK1 | protein_coding |
| rs6947615 | | | | | | | | | | | |
| chr7:3534155:G:A | 0.27 | 0.15 | 1.31 | 0.98 | 1.75 | 1.44 × 10^-1 | REML | Combined | A | SDK1 | protein_coding |
| chr7:3534155:G:A | 0.40 | 0.09 | 1.50 | 1.26 | 1.78 | 3.23 × 10^-6 | FE | European | A | SDK1 | protein_coding |
| chr7:3534155:G:A | -0.38 | 0.22 | 0.69 | 0.44 | 1.06 | 9.02 × 10^-2 | FE | African | A | SDK1 | protein_coding |
| rs28451147 | | | | | | | | | | | |
| chr7:3550467:C:T | 0.27 | 0.15 | 1.31 | 0.98 | 1.75 | 1.44 × 10^-1 | REML | Combined | T | SDK1 | protein_coding, enhancer |
| chr7:3550467:C:T | 0.40 | 0.09 | 1.50 | 1.26 | 1.78 | 3.32 × 10^-6 | FE | European | T | SDK1 | protein_coding, enhancer |
| chr7:3550467:C:T | -0.32 | 0.21 | 0.73 | 0.48 | 1.10 | 1.34 × 10^-1 | FE | African | T | SDK1 | protein_coding, enhancer |
| rs28412607 | | | | | | | | | | | |
| chr7:3551016:T:C | 0.26 | 0.16 | 1.29 | 0.94 | 1.77 | 1.91 × 10^-1 | REML | Combined | C | SDK1 | protein_coding |
| chr7:3551016:T:C | 0.41 | 0.09 | 1.51 | 1.27 | 1.79 | 2.30 × 10^-6 | FE | European | C | SDK1 | protein_coding |
| chr7:3551016:T:C | -0.34 | 0.21 | 0.71 | 0.47 | 1.06 | 9.72 × 10^-2 | FE | African | C | SDK1 | protein_coding |
| rs10236989 | | | | | | | | | | | |
| chr7:3555027:C:T | 0.26 | 0.21 | 1.29 | 0.85 | 1.96 | 3.15 × 10^-1 | REML | Combined | T | SDK1 | protein_coding |
| chr7:3555027:C:T | 0.42 | 0.09 | 1.52 | 1.28 | 1.81 | 2.91 × 10^-6 | FE | European | T | SDK1 | protein_coding |
| chr7:3555027:C:T | -0.31 | 0.21 | 0.73 | 0.48 | 1.10 | 1.33 × 10^-1 | FE | African | T | SDK1 | protein_coding |
| rs142806223 | | | | | | | | | | | |
| chr7:3556215:C:CGAT | 0.29 | 0.18 | 1.34 | 0.94 | 1.91 | 2.04 × 10^-1 | REML | Combined | CGAT | SDK1 | protein_coding |
| chr7:3556215:C:CGAT | 0.43 | 0.09 | 1.53 | 1.29 | 1.83 | 1.74 × 10^-6 | FE | European | CGAT | SDK1 | protein_coding |
| chr7:3556215:C:CGAT | -0.27 | 0.21 | 0.76 | 0.51 | 1.15 | 1.97 × 10^-1 | FE | African | CGAT | SDK1 | protein_coding |
| rs368994553 | | | | | | | | | | | |
| chr7:3556215:C:CGAT | 0.29 | 0.18 | 1.34 | 0.94 | 1.91 | 2.04 × 10^-1 | REML | Combined | CGAT | SDK1 | protein_coding |
| chr7:3556215:C:CGAT | 0.43 | 0.09 | 1.53 | 1.29 | 1.83 | 1.74 × 10^-6 | FE | European | CGAT | SDK1 | protein_coding |
| chr7:3556215:C:CGAT | -0.27 | 0.21 | 0.76 | 0.51 | 1.15 | 1.97 × 10^-1 | FE | African | CGAT | SDK1 | protein_coding |
| rs149369086 | | | | | | | | | | | |
| chr7:3557028:CTAAGT:C | 0.26 | 0.21 | 1.30 | 0.86 | 1.97 | 3.02 × 10^-1 | REML | Combined | C | SDK1 | protein_coding |
| chr7:3557028:CTAAGT:C | 0.42 | 0.09 | 1.52 | 1.28 | 1.82 | 2.96 × 10^-6 | FE | European | C | SDK1 | protein_coding |
| chr7:3557028:CTAAGT:C | -0.46 | 0.25 | 0.63 | 0.38 | 1.04 | 6.89 × 10^-2 | FE | African | C | SDK1 | protein_coding |
| rs375757801 | | | | | | | | | | | |
| chr7:3557028:CTAAGT:C | 0.26 | 0.21 | 1.30 | 0.86 | 1.97 | 3.02 × 10^-1 | REML | Combined | C | SDK1 | protein_coding |
| chr7:3557028:CTAAGT:C | 0.42 | 0.09 | 1.52 | 1.28 | 1.82 | 2.96 × 10^-6 | FE | European | C | SDK1 | protein_coding |
| chr7:3557028:CTAAGT:C | -0.46 | 0.25 | 0.63 | 0.38 | 1.04 | 6.89 × 10^-2 | FE | African | C | SDK1 | protein_coding |
| rs10264275 | | | | | | | | | | | |
| chr7:3557825:A:G | 0.29 | 0.19 | 1.33 | 0.91 | 1.95 | 2.34 × 10^-1 | REML | Combined | G | SDK1 | protein_coding |
| chr7:3557825:A:G | 0.43 | 0.09 | 1.54 | 1.29 | 1.84 | 1.46 × 10^-6 | FE | European | G | SDK1 | protein_coding |
| chr7:3557825:A:G | -0.29 | 0.21 | 0.75 | 0.49 | 1.14 | 1.73 × 10^-1 | FE | African | G | SDK1 | protein_coding |
| rs10215541 | | | | | | | | | | | |
| chr7:3558281:C:T | 0.29 | 0.19 | 1.34 | 0.92 | 1.94 | 2.25 × 10^-1 | REML | Combined | T | SDK1 | protein_coding |
| chr7:3558281:C:T | 0.43 | 0.09 | 1.54 | 1.29 | 1.84 | 1.31 × 10^-6 | FE | European | T | SDK1 | protein_coding |
| chr7:3558281:C:T | -0.28 | 0.21 | 0.75 | 0.49 | 1.14 | 1.83 × 10^-1 | FE | African | T | SDK1 | protein_coding |
| rs10215554 | | | | | | | | | | | |
| chr7:3558650:C:T | 0.29 | 0.19 | 1.34 | 0.92 | 1.94 | 2.27 × 10^-1 | REML | Combined | T | SDK1 | protein_coding |
| chr7:3558650:C:T | 0.44 | 0.09 | 1.55 | 1.30 | 1.84 | 1.18 × 10^-6 | FE | European | T | SDK1 | protein_coding |
| chr7:3558650:C:T | -0.33 | 0.22 | 0.72 | 0.47 | 1.10 | 1.33 × 10^-1 | FE | African | T | SDK1 | protein_coding |
| rs10215632 | | | | | | | | | | | |
| chr7:3558778:C:G | 0.28 | 0.19 | 1.33 | 0.91 | 1.94 | 2.35 × 10^-1 | REML | Combined | G | SDK1 | protein_coding |
| chr7:3558778:C:G | 0.43 | 0.09 | 1.53 | 1.29 | 1.83 | 1.91 × 10^-6 | FE | European | G | SDK1 | protein_coding |
| chr7:3558778:C:G | -0.29 | 0.21 | 0.74 | 0.49 | 1.13 | 1.68 × 10^-1 | FE | African | G | SDK1 | protein_coding |
| rs10216009 | | | | | | | | | | | |
| chr7:3558949:A:G | 0.31 | 0.18 | 1.36 | 0.95 | 1.94 | 1.93 × 10^-1 | REML | Combined | G | SDK1 | protein_coding |
| chr7:3558949:A:G | 0.44 | 0.09 | 1.56 | 1.31 | 1.86 | 8.07 × 10^-7 | FE | European | G | SDK1 | protein_coding |
| chr7:3558949:A:G | -0.25 | 0.21 | 0.78 | 0.51 | 1.18 | 2.32 × 10^-1 | FE | African | G | SDK1 | protein_coding |
| rs17133554 | | | | | | | | | | | |
| chr7:3559476:A:T | 0.28 | 0.20 | 1.33 | 0.90 | 1.97 | 2.49 × 10^-1 | REML | Combined | T | SDK1 | protein_coding |
| chr7:3559476:A:T | 0.44 | 0.09 | 1.55 | 1.30 | 1.84 | 1.32 × 10^-6 | FE | European | T | SDK1 | protein_coding |
| chr7:3559476:A:T | -0.29 | 0.22 | 0.75 | 0.49 | 1.15 | 1.81 × 10^-1 | FE | African | T | SDK1 | protein_coding |
| rs17133555 | | | | | | | | | | | |
| chr7:3559570:C:G | 0.30 | 0.17 | 1.36 | 0.98 | 1.88 | 1.67 × 10^-1 | REML | Combined | G | SDK1 | protein_coding |
| chr7:3559570:C:G | 0.43 | 0.09 | 1.53 | 1.29 | 1.83 | 1.89 × 10^-6 | FE | European | G | SDK1 | protein_coding |
| chr7:3559570:C:G | -0.25 | 0.22 | 0.78 | 0.51 | 1.19 | 2.47 × 10^-1 | FE | African | G | SDK1 | protein_coding |
| rs10227151 | | | | | | | | | | | |
| chr7:3559720:G:C | 0.29 | 0.18 | 1.34 | 0.95 | 1.89 | 1.94 × 10^-1 | REML | Combined | C | SDK1 | protein_coding |
| chr7:3559720:G:C | 0.42 | 0.09 | 1.53 | 1.28 | 1.82 | 1.82 × 10^-6 | FE | European | C | SDK1 | protein_coding |
| chr7:3559720:G:C | -0.21 | 0.21 | 0.81 | 0.54 | 1.22 | 3.16 × 10^-1 | FE | African | C | SDK1 | protein_coding |
| rs10272876 | | | | | | | | | | | |
| chr7:3559948:A:T | 0.31 | 0.17 | 1.36 | 0.98 | 1.90 | 1.67 × 10^-1 | REML | Combined | T | SDK1 | protein_coding |
| chr7:3559948:A:T | 0.44 | 0.09 | 1.55 | 1.30 | 1.84 | 1.16 × 10^-6 | FE | European | T | SDK1 | protein_coding |
| chr7:3559948:A:T | -0.25 | 0.22 | 0.78 | 0.51 | 1.19 | 2.52 × 10^-1 | FE | African | T | SDK1 | protein_coding |
| rs11531493 | | | | | | | | | | | |
| chr7:3561346:G:A | 0.32 | 0.17 | 1.37 | 0.99 | 1.90 | 1.54 × 10^-1 | REML | Combined | A | SDK1 | protein_coding |
| chr7:3561346:G:A | 0.43 | 0.09 | 1.54 | 1.29 | 1.84 | 1.34 × 10^-6 | FE | European | A | SDK1 | protein_coding |
| chr7:3561346:G:A | -0.21 | 0.21 | 0.81 | 0.54 | 1.22 | 3.12 × 10^-1 | FE | African | A | SDK1 | protein_coding |
| rs5022333 | | | | | | | | | | | |
| chr7:3566467:T:C | 0.35 | 0.13 | 1.41 | 1.10 | 1.82 | 7.54 × 10^-2 | REML | Combined | C | SDK1 | protein_coding |
| chr7:3566467:T:C | 0.45 | 0.09 | 1.56 | 1.31 | 1.86 | 7.54 × 10^-7 | FE | European | C | SDK1 | protein_coding |
| chr7:3566467:T:C | -0.14 | 0.20 | 0.87 | 0.59 | 1.28 | 4.77 × 10^-1 | FE | African | C | SDK1 | protein_coding |
| rs10243981 | | | | | | | | | | | |
| chr7:3566706:C:T | 0.31 | 0.18 | 1.37 | 0.96 | 1.96 | 1.84 × 10^-1 | REML | Combined | T | SDK1 | protein_coding |
| chr7:3566706:C:T | 0.45 | 0.09 | 1.57 | 1.31 | 1.87 | 5.95 × 10^-7 | FE | European | T | SDK1 | protein_coding |
| chr7:3566706:C:T | -0.22 | 0.21 | 0.80 | 0.53 | 1.21 | 2.95 × 10^-1 | FE | African | T | SDK1 | protein_coding |
| rs57462209 | | | | | | | | | | | |
| chr7:3571533:G:C | 0.29 | 0.12 | 1.34 | 1.06 | 1.70 | 7.30 × 10^-2 | REML | Combined | C | SDK1 | protein_coding |
| chr7:3571533:G:C | 0.38 | 0.08 | 1.47 | 1.26 | 1.72 | 1.56 × 10^-6 | FE | European | C | SDK1 | protein_coding |
| chr7:3571533:G:C | -0.20 | 0.21 | 0.82 | 0.55 | 1.23 | 3.31 × 10^-1 | FE | African | C | SDK1 | protein_coding |
| rs10085602 | | | | | | | | | | | |
| chr7:3593385:C:T | 0.40 | 0.09 | 1.49 | 1.26 | 1.77 | 3.36 × 10^-6 | FE | Combined | T | SDK1 | protein_coding |
| chr7:3593385:C:T | 0.41 | 0.09 | 1.51 | 1.26 | 1.81 | 5.99 × 10^-6 | FE | European | T | SDK1 | protein_coding |
| chr7:3593385:C:T | 0.07 | 0.26 | 1.08 | 0.65 | 1.80 | 7.75 × 10^-1 | FE | African | T | SDK1 | protein_coding |
| rs10259072 | | | | | | | | | | | |
| chr7:3596826:A:G | 0.39 | 0.08 | 1.48 | 1.26 | 1.73 | 1.88 × 10^-6 | FE | Combined | G | SDK1 | protein_coding, enhancer |
| chr7:3596826:A:G | 0.40 | 0.09 | 1.50 | 1.26 | 1.78 | 5.34 × 10^-6 | FE | European | G | SDK1 | protein_coding, enhancer |
| chr7:3596826:A:G | 0.18 | 0.22 | 1.20 | 0.79 | 1.83 | 3.99 × 10^-1 | FE | African | G | SDK1 | protein_coding, enhancer |
| rs4870896 | | | | | | | | | | | |
| chr8:119203697:A:G | -0.58 | 0.13 | 0.56 | 0.43 | 0.72 | 4.44 × 10^-6 | FE | Combined | A | MAL2 | protein_coding, enhancer |
| chr8:119203697:A:G | -0.57 | 0.13 | 0.56 | 0.44 | 0.72 | 7.06 × 10^-6 | FE | European | A | MAL2 | protein_coding, enhancer |
| rs6995123 | | | | | | | | | | | |
| chr8:119852162:G:A | 0.48 | 0.10 | 1.61 | 1.32 | 1.97 | 2.58 × 10^-6 | FE | Combined | A | DSCC1 | protein_coding, retained_intron, lncRNA |
| chr8:119852162:G:A | 0.48 | 0.13 | 1.61 | 1.26 | 2.07 | 1.74 × 10^-4 | FE | European | A | DSCC1 | protein_coding, retained_intron, lncRNA |
| chr8:119852162:G:A | 0.46 | 0.17 | 1.59 | 1.14 | 2.21 | 5.71 × 10^-3 | FE | African | A | DSCC1 | protein_coding, retained_intron, lncRNA |
| rs7015550 | | | | | | | | | | | |
| chr8:119852406:T:C | 0.53 | 0.11 | 1.70 | 1.38 | 2.09 | 6.66 × 10^-7 | FE | Combined | C | DSCC1 | protein_coding, retained_intron, lncRNA |
| chr8:119852406:T:C | 0.48 | 0.13 | 1.61 | 1.25 | 2.07 | 1.97 × 10^-4 | FE | European | C | DSCC1 | protein_coding, retained_intron, lncRNA |
| chr8:119852406:T:C | 0.61 | 0.19 | 1.83 | 1.27 | 2.66 | 1.36 × 10^-3 | FE | African | C | DSCC1 | protein_coding, retained_intron, lncRNA |
| rs7823109 | | | | | | | | | | | |
| chr8:119863783:C:T | 0.47 | 0.10 | 1.61 | 1.31 | 1.96 | 3.59 × 10^-6 | FE | Combined | T | RN7SL396P, DEPTOR-AS1 | misc_RNA, lncRNA, CTCF_binding_site |
| chr8:119863783:C:T | 0.49 | 0.13 | 1.63 | 1.27 | 2.08 | 1.04 × 10^-4 | FE | European | T | RN7SL396P, DEPTOR-AS1 | misc_RNA, lncRNA, CTCF_binding_site |
| chr8:119863783:C:T | 0.44 | 0.17 | 1.56 | 1.11 | 2.20 | 1.10 × 10^-2 | FE | African | T | RN7SL396P, DEPTOR-AS1 | misc_RNA, lncRNA, CTCF_binding_site |
| rs4316221 | | | | | | | | | | | |
| chr9:29904689:C:T | -0.34 | 0.07 | 0.71 | 0.61 | 0.82 | 4.07 × 10^-6 | FE | Combined | T |  |  |
| chr9:29904689:C:T | -0.30 | 0.08 | 0.74 | 0.63 | 0.87 | 2.63 × 10^-4 | FE | European | T |  |  |
| chr9:29904689:C:T | -0.55 | 0.40 | 0.57 | 0.26 | 1.27 | 4.00 × 10^-1 | REML | African | T |  |  |
| rs9406941 | | | | | | | | | | | |
| chr9:29907925:T:A | -0.36 | 0.08 | 0.70 | 0.60 | 0.81 | 1.49 × 10^-6 | FE | Combined | A |  |  |
| chr9:29907925:T:A | -0.34 | 0.13 | 0.71 | 0.55 | 0.92 | 7.89 × 10^-2 | REML | European | A |  |  |
| chr9:29907925:T:A | -0.57 | 0.32 | 0.57 | 0.30 | 1.05 | 3.24 × 10^-1 | REML | African | A |  |  |
| rs2502097 | | | | | | | | | | | |
| chr9:29913212:T:C | -0.35 | 0.08 | 0.71 | 0.61 | 0.82 | 3.78 × 10^-6 | FE | Combined | C |  |  |
| chr9:29913212:T:C | -0.32 | 0.08 | 0.73 | 0.62 | 0.85 | 9.20 × 10^-5 | FE | European | C |  |  |
| chr9:29913212:T:C | -0.60 | 0.55 | 0.55 | 0.19 | 1.63 | 4.76 × 10^-1 | REML | African | C |  |  |
| rs7847556 | | | | | | | | | | | |
| chr9:29918113:T:A | -0.35 | 0.08 | 0.71 | 0.61 | 0.82 | 3.58 × 10^-6 | FE | Combined | A |  |  |
| chr9:29918113:T:A | -0.32 | 0.08 | 0.73 | 0.62 | 0.85 | 9.73 × 10^-5 | FE | European | A |  |  |
| chr9:29918113:T:A | -0.61 | 0.56 | 0.54 | 0.18 | 1.62 | 4.70 × 10^-1 | REML | African | A |  |  |
| rs145598423 | | | | | | | | | | | |
| chr9:29921085:A:AT | -0.34 | 0.08 | 0.71 | 0.61 | 0.82 | 4.64 × 10^-6 | FE | Combined | AT |  |  |
| chr9:29921085:A:AT | -0.31 | 0.08 | 0.73 | 0.62 | 0.86 | 1.19 × 10^-4 | FE | European | AT |  |  |
| chr9:29921085:A:AT | -0.61 | 0.56 | 0.54 | 0.18 | 1.63 | 4.73 × 10^-1 | REML | African | AT |  |  |
| rs386361211 | | | | | | | | | | | |
| chr9:29921085:A:AT | -0.34 | 0.08 | 0.71 | 0.61 | 0.82 | 4.64 × 10^-6 | FE | Combined | AT |  |  |
| chr9:29921085:A:AT | -0.31 | 0.08 | 0.73 | 0.62 | 0.86 | 1.19 × 10^-4 | FE | European | AT |  |  |
| chr9:29921085:A:AT | -0.61 | 0.56 | 0.54 | 0.18 | 1.63 | 4.73 × 10^-1 | REML | African | AT |  |  |
| rs2502099 | | | | | | | | | | | |
| chr9:29921212:G:A | -0.35 | 0.08 | 0.71 | 0.61 | 0.82 | 4.44 × 10^-6 | FE | Combined | A |  |  |
| chr9:29921212:G:A | -0.32 | 0.08 | 0.73 | 0.62 | 0.86 | 1.14 × 10^-4 | FE | European | A |  |  |
| chr9:29921212:G:A | -0.61 | 0.56 | 0.54 | 0.18 | 1.63 | 4.73 × 10^-1 | REML | African | A |  |  |
| rs2492616 | | | | | | | | | | | |
| chr9:29924649:A:G | -0.36 | 0.08 | 0.70 | 0.60 | 0.81 | 2.28 × 10^-6 | FE | Combined | G |  |  |
| chr9:29924649:A:G | -0.32 | 0.08 | 0.72 | 0.62 | 0.85 | 7.59 × 10^-5 | FE | European | G |  |  |
| chr9:29924649:A:G | -0.62 | 0.55 | 0.54 | 0.18 | 1.58 | 4.63 × 10^-1 | REML | African | G |  |  |
| rs7864973 | | | | | | | | | | | |
| chr9:29924885:A:T | -0.36 | 0.08 | 0.70 | 0.60 | 0.81 | 2.25 × 10^-6 | FE | Combined | T |  |  |
| chr9:29924885:A:T | -0.32 | 0.08 | 0.72 | 0.62 | 0.85 | 7.50 × 10^-5 | FE | European | T |  |  |
| chr9:29924885:A:T | -0.62 | 0.55 | 0.54 | 0.18 | 1.58 | 4.63 × 10^-1 | REML | African | T |  |  |
| rs9408159 | | | | | | | | | | | |
| chr9:29925332:T:C | -0.36 | 0.08 | 0.70 | 0.60 | 0.81 | 1.94 × 10^-6 | FE | Combined | C |  |  |
| chr9:29925332:T:C | -0.33 | 0.08 | 0.72 | 0.61 | 0.84 | 5.34 × 10^-5 | FE | European | C |  |  |
| chr9:29925332:T:C | -0.61 | 0.56 | 0.54 | 0.18 | 1.64 | 4.76 × 10^-1 | REML | African | C |  |  |
| rs2502103 | | | | | | | | | | | |
| chr9:29926017:A:C | -0.36 | 0.08 | 0.69 | 0.60 | 0.81 | 1.50 × 10^-6 | FE | Combined | C |  |  |
| chr9:29926017:A:C | -0.34 | 0.08 | 0.71 | 0.61 | 0.84 | 4.33 × 10^-5 | FE | European | C |  |  |
| chr9:29926017:A:C | -0.61 | 0.57 | 0.54 | 0.18 | 1.65 | 4.76 × 10^-1 | REML | African | C |  |  |
| rs9408160 | | | | | | | | | | | |
| chr9:29927539:G:A | -0.35 | 0.08 | 0.70 | 0.61 | 0.82 | 3.49 × 10^-6 | FE | Combined | A |  |  |
| chr9:29927539:G:A | -0.33 | 0.08 | 0.72 | 0.61 | 0.85 | 7.24 × 10^-5 | FE | European | A |  |  |
| chr9:29927539:G:A | -0.55 | 0.51 | 0.58 | 0.21 | 1.56 | 4.75 × 10^-1 | REML | African | A |  |  |
| rs2099721 | | | | | | | | | | | |
| chr10:13068789:C:T | 0.34 | 0.07 | 1.41 | 1.23 | 1.61 | 7.40 × 10^-7 | FE | Combined | C | CCDC3 | protein_coding |
| chr10:13068789:C:T | 0.33 | 0.08 | 1.39 | 1.20 | 1.61 | 1.48 × 10^-5 | FE | European | C | CCDC3 | protein_coding |
| chr10:13068789:C:T | 0.27 | 0.17 | 1.31 | 0.94 | 1.82 | 1.07 × 10^-1 | FE | African | C | CCDC3 | protein_coding |
| rs36097625 | | | | | | | | | | | |
| chr10:13070107:C:CT | 0.33 | 0.07 | 1.39 | 1.23 | 1.58 | 3.84 × 10^-7 | FE | Combined | C | CCDC3 | protein_coding |
| chr10:13070107:C:CT | 0.31 | 0.07 | 1.37 | 1.19 | 1.57 | 1.18 × 10^-5 | FE | European | C | CCDC3 | protein_coding |
| chr10:13070107:C:CT | 0.30 | 0.16 | 1.35 | 0.98 | 1.87 | 6.66 × 10^-2 | FE | African | C | CCDC3 | protein_coding |
| rs11032349 | | | | | | | | | | | |
| chr11:33708126:A:T | 0.66 | 0.14 | 1.93 | 1.47 | 2.54 | 2.22 × 10^-6 | FE | Combined | T | CD59 | protein_coding, retained_intron |
| chr11:33708126:A:T | 0.57 | 0.14 | 1.77 | 1.35 | 2.34 | 4.50 × 10^-5 | FE | European | T | CD59 | protein_coding, retained_intron |
| rs11213333 | | | | | | | | | | | |
| chr11:110267076:A:C | -0.23 | 0.12 | 0.79 | 0.63 | 1.00 | 1.27 × 10^-1 | REML | Combined | C | RDX | protein_coding, nonsense_mediated_decay |
| chr11:110267076:A:C | -0.37 | 0.08 | 0.69 | 0.59 | 0.81 | 4.90 × 10^-6 | FE | European | C | RDX | protein_coding, nonsense_mediated_decay |
| chr11:110267076:A:C | 0.14 | 0.17 | 1.15 | 0.82 | 1.61 | 4.11 × 10^-1 | FE | African | C | RDX | protein_coding, nonsense_mediated_decay |
| rs34619631 | | | | | | | | | | | |
| chr11:110274650:CAT:C | -0.24 | 0.13 | 0.79 | 0.62 | 1.01 | 1.36 × 10^-1 | REML | Combined | C | RDX | protein_coding, nonsense_mediated_decay |
| chr11:110274650:CAT:C | -0.37 | 0.08 | 0.69 | 0.59 | 0.81 | 3.30 × 10^-6 | FE | European | C | RDX | protein_coding, nonsense_mediated_decay |
| chr11:110274650:CAT:C | 0.15 | 0.17 | 1.16 | 0.83 | 1.62 | 3.88 × 10^-1 | FE | African | C | RDX | protein_coding, nonsense_mediated_decay |
| rs551916313 | | | | | | | | | | | |
| chr11:110274650:CAT:C | -0.24 | 0.13 | 0.79 | 0.62 | 1.01 | 1.36 × 10^-1 | REML | Combined | C | RDX | protein_coding, nonsense_mediated_decay |
| chr11:110274650:CAT:C | -0.37 | 0.08 | 0.69 | 0.59 | 0.81 | 3.30 × 10^-6 | FE | European | C | RDX | protein_coding, nonsense_mediated_decay |
| chr11:110274650:CAT:C | 0.15 | 0.17 | 1.16 | 0.83 | 1.62 | 3.88 × 10^-1 | FE | African | C | RDX | protein_coding, nonsense_mediated_decay |
| rs12226032 | | | | | | | | | | | |
| chr11:110281573:G:A | -0.29 | 0.07 | 0.75 | 0.65 | 0.87 | 7.64 × 10^-5 | FE | Combined | A | RDX | protein_coding, nonsense_mediated_decay |
| chr11:110281573:G:A | -0.37 | 0.08 | 0.69 | 0.59 | 0.81 | 3.34 × 10^-6 | FE | European | A | RDX | protein_coding, nonsense_mediated_decay |
| chr11:110281573:G:A | -0.05 | 0.18 | 0.95 | 0.67 | 1.34 | 7.66 × 10^-1 | FE | African | A | RDX | protein_coding, nonsense_mediated_decay |
| rs12226034 | | | | | | | | | | | |
| chr11:110281587:G:A | -0.26 | 0.07 | 0.77 | 0.67 | 0.88 | 2.22 × 10^-4 | FE | Combined | A | RDX | protein_coding, nonsense_mediated_decay |
| chr11:110281587:G:A | -0.37 | 0.08 | 0.69 | 0.59 | 0.80 | 2.67 × 10^-6 | FE | European | A | RDX | protein_coding, nonsense_mediated_decay |
| chr11:110281587:G:A | 0.05 | 0.16 | 1.05 | 0.76 | 1.45 | 7.66 × 10^-1 | FE | African | A | RDX | protein_coding, nonsense_mediated_decay |
| rs34787877 | | | | | | | | | | | |
| chr11:110285696:G:C | -0.23 | 0.12 | 0.79 | 0.62 | 1.01 | 1.33 × 10^-1 | REML | Combined | C | RDX | protein_coding, nonsense_mediated_decay |
| chr11:110285696:G:C | -0.37 | 0.08 | 0.69 | 0.59 | 0.81 | 4.11 × 10^-6 | FE | European | C | RDX | protein_coding, nonsense_mediated_decay |
| chr11:110285696:G:C | 0.12 | 0.17 | 1.13 | 0.81 | 1.57 | 4.79 × 10^-1 | FE | African | C | RDX | protein_coding, nonsense_mediated_decay |
| rs11213342 | | | | | | | | | | | |
| chr11:110295115:G:A | -0.31 | 0.07 | 0.73 | 0.63 | 0.85 | 2.14 × 10^-5 | FE | Combined | A | RDX | protein_coding, nonsense_mediated_decay, promoter |
| chr11:110295115:G:A | -0.39 | 0.08 | 0.68 | 0.58 | 0.79 | 1.12 × 10^-6 | FE | European | A | RDX | protein_coding, nonsense_mediated_decay, promoter |
| chr11:110295115:G:A | -0.12 | 0.19 | 0.88 | 0.61 | 1.27 | 5.04 × 10^-1 | FE | African | A | RDX | protein_coding, nonsense_mediated_decay, promoter |
| rs12371112 | | | | | | | | | | | |
| chr12:58191755:G:A | 0.12 | 0.20 | 1.13 | 0.77 | 1.66 | 5.63 × 10^-1 | REML | Combined | A |  |  |
| chr12:58191755:G:A | -0.07 | 0.08 | 0.93 | 0.80 | 1.08 | 3.42 × 10^-1 | FE | European | A |  |  |
| chr12:58191755:G:A | 0.94 | 0.20 | 2.57 | 1.73 | 3.83 | 3.02 × 10^-6 | FE | African | A |  |  |
| rs59770361 | | | | | | | | | | | |
| chr12:58205746:G:A | 0.11 | 0.18 | 1.12 | 0.78 | 1.61 | 5.76 × 10^-1 | REML | Combined | A |  |  |
| chr12:58205746:G:A | -0.10 | 0.08 | 0.91 | 0.78 | 1.06 | 2.12 × 10^-1 | FE | European | A |  |  |
| chr12:58205746:G:A | 0.92 | 0.20 | 2.51 | 1.69 | 3.73 | 4.71 × 10^-6 | FE | African | A |  |  |
| rs1195940 | | | | | | | | | | | |
| chr12:131131131:T:C | 0.24 | 0.15 | 1.28 | 0.95 | 1.72 | 1.80 × 10^-1 | REML | Combined | T | ADGRD1 | protein_coding, protein_coding_CDS_not_defined, nonsense_mediated_decay, retained_intron |
| chr12:131131131:T:C | 0.10 | 0.10 | 1.10 | 0.90 | 1.35 | 3.32 × 10^-1 | FE | European | T | ADGRD1 | protein_coding, protein_coding_CDS_not_defined, nonsense_mediated_decay, retained_intron |
| chr12:131131131:T:C | 0.84 | 0.18 | 2.31 | 1.63 | 3.28 | 2.94 × 10^-6 | FE | African | T | ADGRD1 | protein_coding, protein_coding_CDS_not_defined, nonsense_mediated_decay, retained_intron |
| rs9516067 | | | | | | | | | | | |
| chr13:92381663:G:A | 0.31 | 0.07 | 1.36 | 1.19 | 1.54 | 2.90 × 10^-6 | FE | Combined | G | GPC5, GPC5-AS2 | protein_coding, lncRNA |
| chr13:92381663:G:A | 0.30 | 0.07 | 1.35 | 1.17 | 1.55 | 3.15 × 10^-5 | FE | European | G | GPC5, GPC5-AS2 | protein_coding, lncRNA |
| chr13:92381663:G:A | 0.34 | 0.17 | 1.41 | 1.02 | 1.95 | 3.78 × 10^-2 | FE | African | G | GPC5, GPC5-AS2 | protein_coding, lncRNA |
| rs1865997 | | | | | | | | | | | |
| chr15:80199111:T:C | 0.30 | 0.06 | 1.34 | 1.18 | 1.53 | 4.85 × 10^-6 | FE | Combined | C | CTXND1 | protein_coding, enhancer |
| chr15:80199111:T:C | 0.25 | 0.07 | 1.28 | 1.11 | 1.47 | 4.94 × 10^-4 | FE | European | C | CTXND1 | protein_coding, enhancer |
| chr15:80199111:T:C | 0.58 | 0.17 | 1.79 | 1.28 | 2.49 | 5.74 × 10^-4 | FE | African | C | CTXND1 | protein_coding, enhancer |
| rs1865996 | | | | | | | | | | | |
| chr15:80199258:A:G | 0.30 | 0.06 | 1.35 | 1.19 | 1.53 | 4.57 × 10^-6 | FE | Combined | G | CTXND1 | protein_coding, enhancer |
| chr15:80199258:A:G | 0.25 | 0.07 | 1.28 | 1.11 | 1.47 | 4.74 × 10^-4 | FE | European | G | CTXND1 | protein_coding, enhancer |
| chr15:80199258:A:G | 0.55 | 0.17 | 1.73 | 1.24 | 2.40 | 1.17 × 10^-3 | FE | African | G | CTXND1 | protein_coding, enhancer |
| rs6500642 | | | | | | | | | | | |
| chr16:4920801:C:G | 0.24 | 0.19 | 1.27 | 0.87 | 1.84 | 2.82 × 10^-1 | REML | Combined | C | PPL | protein_coding |
| chr16:4920801:C:G | -0.06 | 0.10 | 0.94 | 0.77 | 1.15 | 5.42 × 10^-1 | FE | European | C | PPL | protein_coding |
| chr16:4920801:C:G | 0.90 | 0.19 | 2.46 | 1.70 | 3.56 | 2.06 × 10^-6 | FE | African | C | PPL | protein_coding |
| rs13339040 | | | | | | | | | | | |
| chr16:15711722:T:G | 0.47 | 0.10 | 1.60 | 1.31 | 1.94 | 3.38 × 10^-6 | FE | Combined | G | MYH11, NDE1 | protein_coding, retained_intron, protein_coding_CDS_not_defined, nonsense_mediated_decay |
| chr16:15711722:T:G | 0.47 | 0.12 | 1.61 | 1.26 | 2.04 | 1.13 × 10^-4 | FE | European | G | MYH11, NDE1 | protein_coding, retained_intron, protein_coding_CDS_not_defined, nonsense_mediated_decay |
| chr16:15711722:T:G | 0.45 | 0.17 | 1.57 | 1.12 | 2.20 | 8.68 × 10^-3 | FE | African | G | MYH11, NDE1 | protein_coding, retained_intron, protein_coding_CDS_not_defined, nonsense_mediated_decay |
| rs7205185 | | | | | | | | | | | |
| chr16:15713291:A:G | 0.49 | 0.10 | 1.63 | 1.34 | 1.98 | 1.01 × 10^-6 | FE | Combined | G | MYH11, NDE1 | protein_coding, retained_intron, protein_coding_CDS_not_defined, nonsense_mediated_decay |
| chr16:15713291:A:G | 0.44 | 0.12 | 1.56 | 1.23 | 1.98 | 2.71 × 10^-4 | FE | European | G | MYH11, NDE1 | protein_coding, retained_intron, protein_coding_CDS_not_defined, nonsense_mediated_decay |
| chr16:15713291:A:G | 0.58 | 0.17 | 1.79 | 1.28 | 2.50 | 6.82 × 10^-4 | FE | African | G | MYH11, NDE1 | protein_coding, retained_intron, protein_coding_CDS_not_defined, nonsense_mediated_decay |
| rs34496120 | | | | | | | | | | | |
| chr18:24651297:G:A | 0.22 | 0.18 | 1.25 | 0.87 | 1.77 | 2.91 × 10^-1 | REML | Combined | A | LINC01915 | lncRNA |
| chr18:24651297:G:A | 0.04 | 0.09 | 1.04 | 0.88 | 1.23 | 6.61 × 10^-1 | FE | European | A | LINC01915 | lncRNA |
| chr18:24651297:G:A | 1.14 | 0.23 | 3.12 | 1.97 | 4.94 | 1.26 × 10^-6 | FE | African | A | LINC01915 | lncRNA |
| rs62087910 | | | | | | | | | | | |
| chr18:24652872:A:G | 0.24 | 0.24 | 1.27 | 0.79 | 2.05 | 3.92 × 10^-1 | REML | Combined | G | LINC01915 | lncRNA |
| chr18:24652872:A:G | -0.02 | 0.10 | 0.98 | 0.80 | 1.20 | 8.49 × 10^-1 | FE | European | G | LINC01915 | lncRNA |
| chr18:24652872:A:G | 1.14 | 0.23 | 3.12 | 1.97 | 4.94 | 1.26 × 10^-6 | FE | African | G | LINC01915 | lncRNA |
| rs113042805 | | | | | | | | | | | |
| chr18:60587214:TAA:T | 0.29 | 0.20 | 1.33 | 0.90 | 1.96 | 2.43 × 10^-1 | REML | Combined | T |  |  |
| chr18:60587214:TAA:T | 0.56 | 0.12 | 1.76 | 1.38 | 2.23 | 3.85 × 10^-6 | FE | European | T |  |  |
| chr18:60587214:TAA:T | -0.31 | 0.20 | 0.73 | 0.50 | 1.09 | 1.21 × 10^-1 | FE | African | T |  |  |
| rs80228530 | | | | | | | | | | | |
| chr18:60601053:C:T | 0.33 | 0.34 | 1.39 | 0.71 | 2.71 | 4.05 × 10^-1 | REML | Combined | T |  |  |
| chr18:60601053:C:T | 0.72 | 0.15 | 2.05 | 1.52 | 2.76 | 2.81 × 10^-6 | FE | European | T |  |  |
| chr18:60601053:C:T | -0.59 | 0.24 | 0.56 | 0.35 | 0.89 | 1.43 × 10^-2 | FE | African | T |  |  |
| rs75991968 | | | | | | | | | | | |
| chr18:60602923:A:G | 0.33 | 0.33 | 1.40 | 0.74 | 2.64 | 3.82 × 10^-1 | REML | Combined | G |  |  |
| chr18:60602923:A:G | 0.74 | 0.15 | 2.10 | 1.57 | 2.81 | 5.19 × 10^-7 | FE | European | G |  |  |
| chr18:60602923:A:G | -0.54 | 0.24 | 0.58 | 0.37 | 0.92 | 2.17 × 10^-2 | FE | African | G |  |  |
| rs143279621 | | | | | | | | | | | |
| chr18:60622054:TAAGG:T | 0.29 | 0.29 | 1.33 | 0.75 | 2.36 | 4.01 × 10^-1 | REML | Combined | T | GAD3P | transcribed_unprocessed_pseudogene |
| chr18:60622054:TAAGG:T | 0.72 | 0.15 | 2.05 | 1.52 | 2.77 | 2.35 × 10^-6 | FE | European | T | GAD3P | transcribed_unprocessed_pseudogene |
| chr18:60622054:TAAGG:T | -0.51 | 0.23 | 0.60 | 0.38 | 0.95 | 3.06 × 10^-2 | FE | African | T | GAD3P | transcribed_unprocessed_pseudogene |
| rs550437088 | | | | | | | | | | | |
| chr18:60622054:TAAGG:T | 0.29 | 0.29 | 1.33 | 0.75 | 2.36 | 4.01 × 10^-1 | REML | Combined | T | GAD3P | transcribed_unprocessed_pseudogene |
| chr18:60622054:TAAGG:T | 0.72 | 0.15 | 2.05 | 1.52 | 2.77 | 2.35 × 10^-6 | FE | European | T | GAD3P | transcribed_unprocessed_pseudogene |
| chr18:60622054:TAAGG:T | -0.51 | 0.23 | 0.60 | 0.38 | 0.95 | 3.06 × 10^-2 | FE | African | T | GAD3P | transcribed_unprocessed_pseudogene |
| rs577858875 | | | | | | | | | | | |
| chr18:60622409:T:TA | 0.26 | 0.32 | 1.30 | 0.69 | 2.44 | 4.74 × 10^-1 | REML | Combined | TA | GAD3P | transcribed_unprocessed_pseudogene |
| chr18:60622409:T:TA | 0.70 | 0.15 | 2.02 | 1.50 | 2.71 | 3.17 × 10^-6 | FE | European | TA | GAD3P | transcribed_unprocessed_pseudogene |
| chr18:60622409:T:TA | -0.54 | 0.22 | 0.58 | 0.38 | 0.90 | 1.45 × 10^-2 | FE | African | TA | GAD3P | transcribed_unprocessed_pseudogene |
| rs11388808 | | | | | | | | | | | |
| chr18:60622409:T:TA | 0.26 | 0.32 | 1.30 | 0.69 | 2.44 | 4.74 × 10^-1 | REML | Combined | TA | GAD3P | transcribed_unprocessed_pseudogene |
| chr18:60622409:T:TA | 0.70 | 0.15 | 2.02 | 1.50 | 2.71 | 3.17 × 10^-6 | FE | European | TA | GAD3P | transcribed_unprocessed_pseudogene |
| chr18:60622409:T:TA | -0.54 | 0.22 | 0.58 | 0.38 | 0.90 | 1.45 × 10^-2 | FE | African | TA | GAD3P | transcribed_unprocessed_pseudogene |
| rs11419385 | | | | | | | | | | | |
| chr18:60622409:T:TA | 0.26 | 0.32 | 1.30 | 0.69 | 2.44 | 4.74 × 10^-1 | REML | Combined | TA | GAD3P | transcribed_unprocessed_pseudogene |
| chr18:60622409:T:TA | 0.70 | 0.15 | 2.02 | 1.50 | 2.71 | 3.17 × 10^-6 | FE | European | TA | GAD3P | transcribed_unprocessed_pseudogene |
| chr18:60622409:T:TA | -0.54 | 0.22 | 0.58 | 0.38 | 0.90 | 1.45 × 10^-2 | FE | African | TA | GAD3P | transcribed_unprocessed_pseudogene |
| rs78397733 | | | | | | | | | | | |
| chr18:60622980:G:C | 0.32 | 0.32 | 1.37 | 0.73 | 2.57 | 3.95 × 10^-1 | REML | Combined | C | GAD3P | transcribed_unprocessed_pseudogene |
| chr18:60622980:G:C | 0.71 | 0.15 | 2.04 | 1.51 | 2.77 | 3.90 × 10^-6 | FE | European | C | GAD3P | transcribed_unprocessed_pseudogene |
| chr18:60622980:G:C | -0.51 | 0.23 | 0.60 | 0.38 | 0.94 | 2.46 × 10^-2 | FE | African | C | GAD3P | transcribed_unprocessed_pseudogene |
| rs199894119 | | | | | | | | | | | |
| chr19:22566979:GT:G | 0.90 | 0.19 | 2.45 | 1.68 | 3.56 | 2.86 × 10^-6 | FE | Combined | G |  |  |
| chr19:22566979:GT:G | 0.90 | 0.19 | 2.45 | 1.68 | 3.56 | 2.86 × 10^-6 | FE | European | G |  |  |
| rs60899119 | | | | | | | | | | | |
| chr19:22568524:C:A | 1.01 | 0.19 | 2.73 | 1.88 | 3.98 | 1.54 × 10^-7 | FE | Combined | A |  |  |
| chr19:22568524:C:A | 1.01 | 0.19 | 2.73 | 1.88 | 3.98 | 1.54 × 10^-7 | FE | European | A |  |  |
| rs4933004 | | | | | | | | | | | |
| chr19:22571675:T:C | 0.96 | 0.19 | 2.60 | 1.80 | 3.75 | 3.38 × 10^-7 | FE | Combined | C |  |  |
| chr19:22571675:T:C | 0.96 | 0.19 | 2.60 | 1.80 | 3.75 | 3.38 × 10^-7 | FE | European | C |  |  |
| rs4933005 | | | | | | | | | | | |
| chr19:22571696:C:A | 0.99 | 0.19 | 2.69 | 1.85 | 3.89 | 1.66 × 10^-7 | FE | Combined | A |  |  |
| chr19:22571696:C:A | 0.99 | 0.19 | 2.69 | 1.85 | 3.89 | 1.66 × 10^-7 | FE | European | A |  |  |
| rs59910125 | | | | | | | | | | | |
| chr19:22571719:A:G | 0.94 | 0.19 | 2.57 | 1.79 | 3.71 | 3.90 × 10^-7 | FE | Combined | G |  |  |
| chr19:22571719:A:G | 0.94 | 0.19 | 2.57 | 1.79 | 3.71 | 3.90 × 10^-7 | FE | European | G |  |  |
| rs28770002 | | | | | | | | | | | |
| chr19:22574453:C:T | 0.94 | 0.19 | 2.56 | 1.77 | 3.70 | 5.10 × 10^-7 | FE | Combined | T |  |  |
| chr19:22574453:C:T | 0.94 | 0.19 | 2.56 | 1.77 | 3.70 | 5.10 × 10^-7 | FE | European | T |  |  |
| rs28787279 | | | | | | | | | | | |
| chr19:22574483:C:T | 0.91 | 0.19 | 2.49 | 1.73 | 3.59 | 1.03 × 10^-6 | FE | Combined | T |  |  |
| chr19:22574483:C:T | 0.91 | 0.19 | 2.49 | 1.73 | 3.59 | 1.03 × 10^-6 | FE | European | T |  |  |
| rs8110963 | | | | | | | | | | | |
| chr19:22579782:A:C | 0.80 | 0.17 | 2.23 | 1.60 | 3.12 | 2.68 × 10^-6 | FE | Combined | C |  |  |
| chr19:22579782:A:C | 0.80 | 0.17 | 2.23 | 1.60 | 3.12 | 2.68 × 10^-6 | FE | European | C |  |  |
| rs10423182 | | | | | | | | | | | |
| chr19:22580332:C:G | 0.79 | 0.17 | 2.20 | 1.57 | 3.07 | 3.99 × 10^-6 | FE | Combined | G |  |  |
| chr19:22580332:C:G | 0.79 | 0.17 | 2.20 | 1.57 | 3.07 | 3.99 × 10^-6 | FE | European | G |  |  |
| rs147537414 | | | | | | | | | | | |
| chr19:22582475:ATT:A | 0.81 | 0.17 | 2.24 | 1.60 | 3.13 | 2.41 × 10^-6 | FE | Combined | A |  |  |
| chr19:22582475:ATT:A | 0.81 | 0.17 | 2.24 | 1.60 | 3.13 | 2.41 × 10^-6 | FE | European | A |  |  |
| rs535381996 | | | | | | | | | | | |
| chr19:22585665:G:T | 0.79 | 0.17 | 2.20 | 1.57 | 3.07 | 3.88 × 10^-6 | FE | Combined | T |  |  |
| chr19:22585665:G:T | 0.79 | 0.17 | 2.20 | 1.57 | 3.07 | 3.88 × 10^-6 | FE | European | T |  |  |
| rs553808119 | | | | | | | | | | | |
| chr19:22585666:G:T | 0.79 | 0.17 | 2.20 | 1.57 | 3.07 | 3.88 × 10^-6 | FE | Combined | T |  |  |
| chr19:22585666:G:T | 0.79 | 0.17 | 2.20 | 1.57 | 3.07 | 3.88 × 10^-6 | FE | European | T |  |  |
| rs199702934 | | | | | | | | | | | |
| chr19:22585668:C:T | 0.79 | 0.17 | 2.20 | 1.57 | 3.07 | 3.88 × 10^-6 | FE | Combined | T |  |  |
| chr19:22585668:C:T | 0.79 | 0.17 | 2.20 | 1.57 | 3.07 | 3.88 × 10^-6 | FE | European | T |  |  |
| rs11083715 | | | | | | | | | | | |
| chr19:43686219:C:T | 0.18 | 0.17 | 1.20 | 0.85 | 1.69 | 3.55 × 10^-1 | REML | Combined | T |  | transcribed_unprocessed_pseudogene |
| chr19:43686219:C:T | 0.05 | 0.07 | 1.05 | 0.91 | 1.21 | 5.13 × 10^-1 | FE | European | T |  | transcribed_unprocessed_pseudogene |
| chr19:43686219:C:T | 0.88 | 0.18 | 2.40 | 1.68 | 3.45 | 1.82 × 10^-6 | FE | African | T |  | transcribed_unprocessed_pseudogene |
| rs11083716 | | | | | | | | | | | |
| chr19:43686510:G:A | 0.19 | 0.17 | 1.20 | 0.87 | 1.68 | 3.33 × 10^-1 | REML | Combined | A |  | transcribed_unprocessed_pseudogene |
| chr19:43686510:G:A | 0.05 | 0.07 | 1.06 | 0.91 | 1.22 | 4.59 × 10^-1 | FE | European | A |  | transcribed_unprocessed_pseudogene |
| chr19:43686510:G:A | 0.87 | 0.18 | 2.39 | 1.66 | 3.43 | 2.46 × 10^-6 | FE | African | A |  | transcribed_unprocessed_pseudogene |
| rs11083718 | | | | | | | | | | | |
| chr19:43688323:G:A | 0.27 | 0.17 | 1.31 | 0.93 | 1.85 | 1.93 × 10^-1 | REML | Combined | A |  | transcribed_unprocessed_pseudogene, lncRNA |
| chr19:43688323:G:A | 0.10 | 0.08 | 1.10 | 0.94 | 1.29 | 2.19 × 10^-1 | FE | European | A |  | transcribed_unprocessed_pseudogene, lncRNA |
| chr19:43688323:G:A | 0.86 | 0.18 | 2.36 | 1.65 | 3.37 | 2.83 × 10^-6 | FE | African | A |  | transcribed_unprocessed_pseudogene, lncRNA |
| rs6010720 | | | | | | | | | | | |
| chr20:64093106:G:A | 0.46 | 0.09 | 1.59 | 1.33 | 1.90 | 3.10 × 10^-7 | FE | Combined | G | OPRL1 | protein_coding, lncRNA, CTCF_binding_site |
| chr20:64093106:G:A | 0.46 | 0.10 | 1.59 | 1.31 | 1.91 | 1.67 × 10^-6 | FE | European | G | OPRL1 | protein_coding, lncRNA, CTCF_binding_site |
| chr20:64093106:G:A | 0.49 | 0.27 | 1.63 | 0.97 | 2.75 | 6.58 × 10^-2 | FE | African | G | OPRL1 | protein_coding, lncRNA, CTCF_binding_site |
| rs6010721 | | | | | | | | | | | |
| chr20:64099325:T:G | 0.42 | 0.09 | 1.53 | 1.28 | 1.82 | 2.51 × 10^-6 | FE | Combined | T | OPRL1, MYT1, NPBWR2 | protein_coding, lncRNA |
| chr20:64099325:T:G | 0.42 | 0.10 | 1.52 | 1.26 | 1.84 | 1.33 × 10^-5 | FE | European | T | OPRL1, MYT1, NPBWR2 | protein_coding, lncRNA |
| chr20:64099325:T:G | 0.48 | 0.24 | 1.62 | 1.01 | 2.60 | 4.46 × 10^-2 | FE | African | T | OPRL1, MYT1, NPBWR2 | protein_coding, lncRNA |
| rs6089789 | | | | | | | | | | | |
| chr20:64100639:T:C | 0.43 | 0.09 | 1.54 | 1.28 | 1.84 | 3.56 × 10^-6 | FE | Combined | T | OPRL1, MYT1, NPBWR2 | protein_coding, lncRNA |
| chr20:64100639:T:C | 0.44 | 0.10 | 1.55 | 1.28 | 1.87 | 7.43 × 10^-6 | FE | European | T | OPRL1, MYT1, NPBWR2 | protein_coding, lncRNA |
| chr20:64100639:T:C | 0.39 | 0.31 | 1.48 | 0.81 | 2.71 | 2.06 × 10^-1 | FE | African | T | OPRL1, MYT1, NPBWR2 | protein_coding, lncRNA |
| rs6062630 | | | | | | | | | | | |
| chr20:64103792:C:T | 0.42 | 0.09 | 1.52 | 1.27 | 1.82 | 4.26 × 10^-6 | FE | Combined | C | OPRL1, NPBWR2, MYT1 | protein_coding, lncRNA |
| chr20:64103792:C:T | 0.41 | 0.10 | 1.51 | 1.25 | 1.83 | 2.16 × 10^-5 | FE | European | C | OPRL1, NPBWR2, MYT1 | protein_coding, lncRNA |
| chr20:64103792:C:T | 0.49 | 0.27 | 1.63 | 0.97 | 2.75 | 6.58 × 10^-2 | FE | African | C | OPRL1, NPBWR2, MYT1 | protein_coding, lncRNA |
| rs4809397 | | | | | | | | | | | |
| chr20:64104214:C:T | 0.42 | 0.09 | 1.52 | 1.28 | 1.82 | 3.24 × 10^-6 | FE | Combined | C | OPRL1, NPBWR2, MYT1 | protein_coding, lncRNA, enhancer |
| chr20:64104214:C:T | 0.41 | 0.10 | 1.51 | 1.25 | 1.83 | 1.92 × 10^-5 | FE | European | C | OPRL1, NPBWR2, MYT1 | protein_coding, lncRNA, enhancer |
| chr20:64104214:C:T | 0.49 | 0.25 | 1.63 | 0.99 | 2.68 | 5.37 × 10^-2 | FE | African | C | OPRL1, NPBWR2, MYT1 | protein_coding, lncRNA, enhancer |
| rs7291786 | | | | | | | | | | | |
| chr22:23998350:C:T | 0.44 | 0.09 | 1.55 | 1.29 | 1.85 | 2.27 × 10^-6 | FE | Combined | T | GSTT4 | nonsense_mediated_decay, retained_intron, protein_coding |
| chr22:23998350:C:T | 0.46 | 0.10 | 1.58 | 1.29 | 1.94 | 8.47 × 10^-6 | FE | European | T | GSTT4 | nonsense_mediated_decay, retained_intron, protein_coding |
| chr22:23998350:C:T | 0.23 | 0.20 | 1.26 | 0.84 | 1.88 | 2.60 × 10^-1 | FE | African | T | GSTT4 | nonsense_mediated_decay, retained_intron, protein_coding |
