# Supplementary material for: Diversity in genetic risk of recurrent stroke: a genome-wide association study meta-analysis
Source: Front Stroke. 2024 Feb 21;3:1338636. doi: 10.3389/fstro.2024.1338636 (PMC12802773; doi:10.3389/fstro.2024.1338636)
Supplement: Supplementary file 2 [file Table_2.DOCX]

| rsid | Effect.Allele | odds_ratio | conf.low | conf.high | p.value | gene | feature_type | ASGC | REGARDS_AF | REGARDS_EU | SAHLSIS | VISP_EU | VISP_AF |
| --- | --- | --- | --- | --- | --- | --- | --- | --- | --- | --- | --- | --- | --- |
| rs1032996 | C | 2.07 | 1.53 | 2.79 | 1.85e-06 |  | RegulatoryFeature | 0.27 | 0.13 | - | - | - | - |
| rs6692497 | G | 1.51 | 1.27 | 1.79 | 1.81e-06 |  |  | 0.13 | 0.15 | 0.12 | 0.3 | 0.25 | 0.29 |
| rs16842987 | A | 3.63 | 2.11 | 6.25 | 3.24e-06 |  |  | 0.02 | 0.07 | - | - | - | - |
| rs62169505 | G | 1.39 | 1.22 | 1.58 | 5.51e-07 |  | Transcript | 0.33 | 0.27 | 0.36 | 0.61 | 0.58 | 0.41 |
| rs688931 | G | 1.52 | 1.27 | 1.81 | 3.91e-06 | PELO-AS1 | Transcript | 0.09 | 0.11 | 0.18 | 0.31 | 0.29 | 0.2 |
| rs61408734 | T | 1.98 | 1.50 | 2.60 | 1.34e-06 | PPARGC1B | Transcript | - | 0.17 | - | 0.06 | 0.1 | 0.31 |
| rs10259072 | G | 1.48 | 1.26 | 1.73 | 1.88e-06 | SDK1 | Transcript, RegulatoryFeature | 0.12 | 0.15 | 0.23 | 0.41 | 0.39 | 0.29 |
| rs4870896 | A | 0.56 | 0.43 | 0.72 | 4.44e-06 | MAL2 | Transcript, RegulatoryFeature | 0.18 | - | 0.1 | 0.12 | 0.15 | - |
| rs7015550 | C | 1.70 | 1.38 | 2.09 | 6.66e-07 | DSCC1 | Transcript | 0.04 | 0.18 | 0.06 | 0.15 | 0.13 | 0.35 |
| rs9406941 | A | 0.70 | 0.60 | 0.81 | 1.49e-06 |  |  | 0.29 | 0.36 | 0.29 | 0.47 | 0.51 | 0.66 |
| rs36097625 | C | 1.39 | 1.23 | 1.58 | 3.84e-07 | CCDC3 | Transcript | 0.48 | 0.49 | 0.48 | 0.68 | 0.72 | 0.72 |
| rs11032349 | T | 1.93 | 1.47 | 2.54 | 2.22e-06 | CD59 | Transcript | - | - | 0.11 | - | 0.21 | - |
| rs9516067 | G | 1.36 | 1.19 | 1.54 | 2.90e-06 | GPC5, GPC5-AS2 | Transcript | 0.4 | 0.5 | 0.5 | 0.69 | 0.74 | 0.75 |
| rs1865996 | G | 1.35 | 1.19 | 1.53 | 4.57e-06 | CTXND1 | Transcript, RegulatoryFeature | 0.33 | 0.46 | 0.43 | 0.66 | 0.65 | 0.67 |
| rs7205185 | G | 1.63 | 1.34 | 1.98 | 1.01e-06 | MYH11, NDE1 | Transcript | 0.08 | 0.34 | 0.08 | 0.14 | 0.18 | 0.62 |
| rs60899119 | A | 2.73 | 1.88 | 3.98 | 1.54e-07 |  |  | 0.05 | - | - | 0.11 | - | - |
| rs6010720 | G | 1.59 | 1.33 | 1.90 | 3.10e-07 | OPRL1 | Transcript, RegulatoryFeature | 0.31 | 0.09 | 0.09 | 0.16 | 0.15 | 0.13 |
| rs7291786 | T | 1.55 | 1.29 | 1.85 | 2.27e-06 | GSTT4 | Transcript | 0.08 | 0.18 | 0.11 | 0.21 | 0.29 | 0.35 |
| ASGC is the Australian Stroke Genetics Collaboration. REGARDS refers to the Reasons for Geographic and Racial Differences in Stroke. SAHLSIS is the Sahlgrenska Academy Study on Ischemic Stroke. VISP refers to the Vitamin Interventio for Stroke Prevention trial. AF refers to African ancestry, while EU is European Ancestry. Gene Assembly is GRChr38. | | | | | | | | | | | | | |
