# Supplementary material for: Diversity in genetic risk of recurrent stroke: a genome-wide association study meta-analysis
Source: Front Stroke. 2024 Feb 21;3:1338636. doi: 10.3389/fstro.2024.1338636 (PMC12802773; doi:10.3389/fstro.2024.1338636)
Supplement: Supplementary file 3 [file Data_Sheet_1.DOCX]

Genotyping and Quality Control Methods

All cohorts underwent the following quality control protocol: The genotyped data underwent strict quality control measures using Plink (1) that filtered out SNPs as follows: 1) missing call rate *>* 2%, 2) Mendelian errors in control trios, 3) deviation from Hardy-Weinberg equilibrium in controls, 4) discordant calls in duplicate samples, 5) sex differences in allele frequency or heterozygosity, 6) and minor allele frequency *<* 0.05 in line with previously published recommendations. (2) Cohort-specific methods are noted below.

## Australian Stroke Genetics Collaboration (ASGC) (3,4)

AUST genotyped their samples using the Illumina HumanHap610-Quad array. Quality control excluded SNPS not present in cases and control samples, call rate < 0.95, deviation of Hardy-Weinberg equilibrium (p<1x10^-6^) or a minor allele frequency <0.01. Individual samples were excluded due to low call rates (<0.95), gender discrepancy, unexpected relatedness or evidence of non-European ancestry. Missing genotyped SNPs were imputed via the 1000 Genomes version 3 reference panel (5) with the Minimac Imputation procedure. (6) After all quality control steps, 7,500,572 SNPs remained.

## Reasons for Geographic and Racial Differences in Stroke (REGARDS) (7)

The genotyping, quality control, and imputation methodologies of the REGARDS study have been described previously (8). Briefly, genome-wide genotyping was performed using Illumina Infinium Multi-Ethnic AMR/AFR Extended BeadChip arrays (MEGA, Illumina, Inc., San Diego, CA). Participants were excluded with call rates less than 95%, if they were internal duplicates, had sex mismatches, or if they were outliers on principal component analysis outside of six standard deviations. Variants were excluded if they were multi-allelic, if the strands were ambiguous or inconsistent, if the variants were located on sex chromosomes, or were in violation of Hardy Weinberg equilibrium (HWE < 1E-05 for White participants, HWE < 1E-12 for Black participants). Filtered genotype calls were imputed to the NHLBI Trans-Omics for Precision Medicine (TOPMed) release 2 (Freeze 8) reference panel. Post-imputation QC excluded variants with imputation quality scores (rsq) < 0.3 and a minor allele frequency (MAF) < 0.05.

## Sahlgrenska Academy Study on Ischemic Stroke (SAHLSIS) (9,10)

Genotyping was performed on the Illumin Human OmniExpressExome BeadChip version 1.0 or 1.1 with the iScan system. Genotypes were called using the Autocall algorithm then compared to the 1000 Genomes sample of European ancestry at the Board Institute (Boston, USA) between August 2012 and April 2013. Quality control consisted of excluding SNPs with a call rate < 0.95 and Hardy-Weinberg Equilibrium (p<10^-6^). Samples with inbreeding coefficients of -0.2 to 0.2 or call rate <0.95 were also excluded (11). We increased the number of SNPs with imputation via the 1000 Genomes version reference panel (5) with the Minimac Imputation procedure. (6) After all quality control steps, 7,422,449 SNPs remained.

## Vitamin Intervention for Stroke Prevention (VISP) (12)

The Center for Inherited Disease Research at Johns Hopkins University performed genotyping on the Illumina HumanOmni1-Quad-v1 array (Illumina, Inc.) We increased the number of SNP with genetic imputation via the TOPMed Imputation server (13,14), which implements the Minimac Imputation procedure. (6) After filtering out imputed SNPs with poor imputation quality (*r*^2^ *<* 0*.*80) and MAFs *<* 0*.*05, the final count of SNPs came to 6,392,746 for both African and European ancestries. We calculated the first 10 principal components with the KING software for population structure. (15)

# Citations

1. Chang CC, Chow CC, Tellier LC, Vattikuti S, Purcell SM, Lee JJ, et al. Second-generation PLINK: rising to the challenge of larger and richer datasets. Gigascience. 2015; 4: 7. 2015.

2. Integrating common and rare genetic variation in diverse human populations. Nature. 2010 Sep 2;467(7311):52–8.

3. Holliday EG, Maguire JM, Evans TJ, Koblar SA, Jannes J, Sturm JW, et al. Common variants at 6p21.1 are associated with large artery atherosclerotic stroke. Nat Genet. 2012 Oct;44(10):1147–51.

4. Maguire J, Thakkinstian A, Levi C, Lincz L, Bisset L, Sturm J, et al. Impact of COX-2 rs5275 and rs20417 and GPIIIa rs5918 polymorphisms on 90-day ischemic stroke functional outcome: a novel finding. J Stroke Cerebrovasc Dis Off J Natl Stroke Assoc. 2011;20(2):134–44.

5. Auton A, Abecasis GR, Altshuler DM, Durbin RM, Abecasis GR, Bentley DR, et al. A global reference for human genetic variation. Nature. 2015 Oct;526(7571):68–74.

6. Fuchsberger C, Abecasis GR, Hinds DA. minimac2: faster genotype imputation. Bioinformatics. 2015 Mar 1;31(5):782–4.

7. Howard VJ, Cushman M, Pulley L, Gomez CR, Go RC, Prineas RJ, et al. The reasons for geographic and racial differences in stroke study: objectives and design. Neuroepidemiology. 2005;25(3):135–43.

8. Armstrong ND, Srinivasasainagendra V, Patki A, Tanner RM, Hidalgo BA, Tiwari HK, et al. Genetic Contributors of Incident Stroke in 10,700 African Americans With Hypertension: A Meta-Analysis From the Genetics of Hypertension Associated Treatments and Reasons for Geographic and Racial Differences in Stroke Studies. Front Genet. 2021;12:781451.

9. Jood K, Ladenvall C, Rosengren A, Blomstrand C, Jern C. Family history in ischemic stroke before 70 years of age: The Sahlgrenska academy study on ischemic stroke. Stroke. 2005 Jul;36(7):1383–7.

10. Pedersen A, Redfors P, Lundberg L, Gils A, Declerck PJ, Nilsson S, et al. Haemostatic biomarkers are associated with long-term recurrent vascular events after ischaemic stroke. Thromb Haemost. 2016;116(9):537–43.

11. Söderholm M, Almgren P, Jood K, Stanne TM, Olsson M, Ilinca A, et al. Exome array analysis of ischaemic stroke: results from a southern Swedish study. Eur J Neurol. 2016;23(12):1722–8.

12. Toole JF. Vitamin intervention for stroke prevention. J Neurol Sci. 2002 Nov 15;203–204:121–4.

13. Taliun D, Harris DN, Kessler MD, Carlson J, Szpiech ZA, Torres R, et al. Sequencing of 53,831 diverse genomes from the NHLBI TOPMed Program. Nature. 2021 Feb 11;590(7845):290–9.

14. Das S, Forer L, Schönherr S, Sidore C, Locke AE, Kwong A, et al. Next-generation genotype imputation service and methods. Nat Genet. 2016 Jan;48(10):1284–7.

15. Manichaikul A, Mychaleckyj JC, Rich SS, Daly K, Sale M, Chen WM. Robust relationship inference in genome-wide association studies. Bioinformatics. 2010;26(22):2867–73.
